# Applicability of Quantum Dots in Breast Cancer Diagnostic and Therapeutic Modalities—A State-of-the-Art Review

**DOI:** 10.3390/nano14171424

**Published:** 2024-08-31

**Authors:** Dominika Kunachowicz, Karolina Kłosowska, Natalia Sobczak, Marta Kepinska

**Affiliations:** 1 Department of Pharmaceutical Biochemistry, Faculty of Pharmacy, Wroclaw Medical University, Borowska 211A, 50-556 Wroclaw, Poland; 2 Students’ Scientific Association at the Department of Pharmaceutical Biochemistry (SKN No. 214), Faculty of Pharmacy, Wroclaw Medical University, Borowska 211A, 50-556 Wroclaw, Poland; karolina.klosowska@student.umw.edu.pl; 3 Students’ Scientific Association of Biomedical and Environmental Analyses (SKN No. 85), Faculty of Pharmacy, Wroclaw Medical University, Borowska 211A, 50-556 Wroclaw, Poland; natalia.sobczak@student.umw.edu.pl

**Keywords:** breast cancer, quantum dots, nanomedicine, cancer treatment, targeted drug delivery

## Abstract

The increasing incidence of breast cancers (BCs) in the world population and their complexity and high metastatic ability are serious concerns for healthcare systems. Despite the significant progress in medicine made in recent decades, the efficient treatment of invasive cancers still remains challenging. Chemotherapy, a fundamental systemic treatment method, is burdened with severe adverse effects, with efficacy limited by resistance development and risk of disease recurrence. Also, current diagnostic methods have certain drawbacks, attracting attention to the idea of developing novel, more sensitive detection and therapeutic modalities. It seems the solution for these issues can be provided by nanotechnology. Particularly, quantum dots (QDs) have been extensively evaluated as potential targeted drug delivery vehicles and, simultaneously, sensing and bioimaging probes. These fluorescent nanoparticles offer unlimited possibilities of surface modifications, allowing for the attachment of biomolecules, such as antibodies or proteins, and drug molecules, among others. In this work, we discuss the potential applicability of QDs in breast cancer diagnostics and treatment in light of the current knowledge. We begin with introducing the molecular and histopathological features of BCs, standard therapeutic regimens, and current diagnostic methods. Further, the features of QDs, along with their uptake, biodistribution patterns, and cytotoxicity, are described. Based on the reports published in recent years, we present the progress in research on possible QD use in improving BC diagnostics and treatment efficacy as chemotherapeutic delivery vehicles and photosensitizing agents, along with the stages of their development. We also address limitations and open questions regarding this topic.

## 1. Introduction

The efficient treatment of cancers remains a challenge for modern medicine, given a constant rise in the number of diagnosed cases. This figure worldwide in 2020 exceeded 2 million, and its further increase is expected over the coming years [1]. The most common malignancy in women, characterized by the highest mortality rate, is breast cancer (BC). It is a particularly heterogeneous disease at multiple levels—genetic, epigenetic, transcriptomic, and proteomic [2]. BC is associated mainly with the female population; in males it occurs rarely, constituting less than 1% of all cases [3]. The incidence of BC in developed countries is almost twice as high as in developing ones, which can be attributed to many factors, including stress, environmental pollution, increased alcohol consumption, and dietary patterns. In addition, aging of the general population and increased lifespan resulting from improved medical care promotes the accumulation of DNA mutations and cancer development over time—it is estimated that the incidence of BC will increase by almost 50% by 2040 [4,5].

Early detection of BC is of key importance, enabling immediate implementation of treatment and increasing the chance of therapeutic success. The conventional method for BC screening is mammography, but it is only able to identify malignancies already grown to a specific size. Up to 20% of BCs are neither detectable on mammograms nor properly identified [6]. Also, in the case of needle biopsies there is a relatively high percentage (up to 10%) of false negative results, delaying the moment of correct diagnosis [7]. Serum biomarkers, although they provide insight into disease progression and/or response to therapy, are not as established in BC diagnosis. Additionally, in its early stages the levels of certain proteins (antigens, enzymes, etc.) in circulation are too low to reach the detection limit of current analytical techniques. All of the above contributes to the fact that BC remains underdiagnosed—when it comes to early cancer diagnosis and monitoring, standard cancer detection approaches are not sufficient [8]. Therefore, there is a need to develop novel, more sensitive diagnostic methods to greatly enhance the detection rates of such biomolecules, even at low levels, as well as to perform high-resolution imaging of tumors and single cells. Also, treatment of BC is impeded by frequent complications, such as metastases and resistance to conventional anticancer drugs, leading to therapeutic inefficacy. Moreover, a large number of medications of current therapeutic regimens lack the specificity towards cancer cells, and their use is accompanied by severe adverse effects [9]. Thus, to increase the rate of therapeutic success among BC patients, these limitations must be addressed and overcome through the development of new, cancer cell-selective therapeutic strategies with a more favorable safety profile and lower systemic toxicity than standard chemotherapy.

Decades of progress in nanotechnology have greatly impacted various fields of science and engineering, including biomedicine. Constantly, novel synthetic nanomaterials are being developed to be used as modern sensors in disease diagnostics, drug delivery, and bioimaging [10]. Different nanoscale-based technologies are used by various branches of medicine and pharmacy, and cancer research is nowadays one of the leading directions [11,12]. One particular type of innovative nanomaterial that is continuously gaining in importance is quantum dots (QDs), defined as spherical nanocrystals of typical diameter between 2 and 10 nm [13]. QDs absorb UV light in a broad range and emit fluorescence light at specific wavelengths, depending on their composition and size. Notably, several types of QD, when excited by a single external light source, produces multicolored fluorescence, allowing one to label multiple targets simultaneously within a single sample. Contrary to the broad excitation spectra of QDs, their emission bands are narrow and symmetric. QDs efficiently convert absorbed photons into emitted fluorescence, which is bright and stable. This property, along with ease of surface functionalization, enables sensitive detection of target molecules or cells, even at low concentrations, making QDs valuable tools for detecting biomarkers or studying rare cell populations in complex biological samples [14,15].

As a result of wide-ranging studies regarding QD applicability in cancer diagnosis and therapy, including BC, a considerable number of papers are being published [16]. Recent literature contains numerous reports of successful QD use against BC based on cell culture studies, as well as advances in their potential role and relevance as diagnostic tools in this disease. Therefore, in this review we aim to gather and present the current state of knowledge and advancements concerning the applicability of QDs in both the diagnosis and therapy of BC.

## 2. Breast Cancers: Molecular Features, Histopathological Characteristics, and Clinical Management

### 2.1. Classification of Breast Cancers

**Figure 1:**
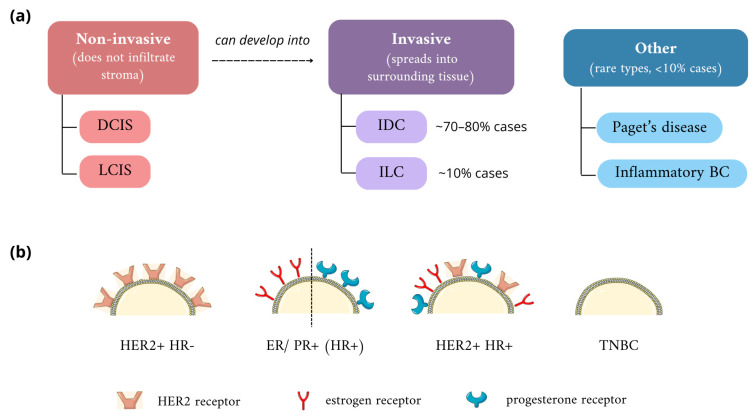
(**a**) General histological and (**b**) molecular classification of breast cancers. Created with Servier Medical Art (https://smart.servier.com/).

According to traditional classification, based on the location of the lesion and clinicopathological features, BC is divided into two main categories: non-invasive (in situ, also called non-infiltrative) cancers, when atypical cells do not penetrate into stroma, and invasive (infiltrative) (Figure 1). The first group includes ductal carcinoma in situ (DCIS) and lobular carcinoma in situ (LCIS); notably, both types can transform into invasive forms, infiltrating surrounding tissues [17]. DCIS is limited to the milk ducts and does not cross the basal membrane. According to the American Cancer Society, it accounts for every 1 in 5 newly diagnosed breast cancer cases [18]. In the vast majority of cases, it is detected accidentally, and death due to this type of cancer is very rare, but the heterogeneous microenvironment of DCIS increases the risk of developing the invasive type—infiltrating ductal carcinoma (IDC) [19,20]. LCIS, representing 1–2% of BCs, is mostly found in premenopausal middle-aged women and is often regarded as a pre-cancerous lesion, which may progress into invasive lobular carcinoma (ILC) [21]. Typically, LCIS exhibits hormone receptor positivity; therefore, hormone therapy based on antiestrogens can be used as a preventive factor, reducing the risk of developing invasive cancer, along with surgical removal of the tumor. Other types, such as Paget’s disease or inflammatory BC, are also distinguished. The latter is characterized by an unfavorable prognosis due to rapid growth [22,23].

Among invasive breast cancers, over 20 histological variants are distinguished, differing between each other in terms of clinical manifestation, biomarker profiles, and gene expression signatures. IDC of no special type (NST) is the most prevalent kind of invasive cancer, covering approximately 70–80% of all cases, with systemic therapy often being ineffective [24]. Invasive lobular carcinoma, forming narrow, banded infiltrations, is the second most popular and accounts for approximately 10%. Due to the pattern of its spread, it is difficult to diagnose by mammography [25]. WHO histological classification also considers cancer cell type (e.g., apocrine carcinomas), immunohistochemical features, extracellular secretion (mucous carcinomas), tissue origin (papillary, neuroendocrine, epithelial-myoepithelial), and architectural features, and is evolving over time due to the broadening of medical knowledge [26]. Conventional prognostic parameters include tumor size, histological grade reflecting the level of differentiation, lymph node status, and occurrence of lymphovascular invasion, but new concepts have emerged that propose drawing more attention to genetic features and the tumor microenvironment [27].

Molecular classification is based on the expression of specific biomarkers: estrogen receptor (ER), progesterone receptor (PR), together known as hormone receptors (HR), and human epidermal growth factor receptor 2 (HER2). A tumor is regarded as ER- or PR-positive when at least 1% of its cells show expression of the particular receptor proven by immunohistochemical staining. Therefore, BC cases are categorized into four subtypes: ER+, PR+, HER2+, and triple-negative breast cancer (TNBC), which does not express any of the aforementioned receptors [28,29]. Distinction between these subtypes allows for the choice of therapy with the highest success probability, e.g., ER expression status is a strong predictive factor of hormonal therapy efficacy, while HER2+ carcinomas present a favorable response to targeted therapies against this protein, such as trastuzumab [27].

Another important, clinically evaluated feature of breast cancer is expression of the nuclear Ki67 protein, regarded as a cell proliferation marker since it is abundant in proliferating cells (G1 and S phases of the cell cycle) and is not detectable in dormant cells (G0 and early G1 phase). The number of Ki67+ tumor cells, expressed as their percentage, are used to stratify patients into groups of good and poor prognosis (the higher the Ki67 index, the worse the prognosis). This parameter is also used to aid in defining the intrinsic subtypes of invasive BC as luminal A, which shows low expression of Ki67 (below 14%), and luminal B [27].

### 2.2. Breast Cancer Heterogeneity and Its Basis

**Figure 2:**
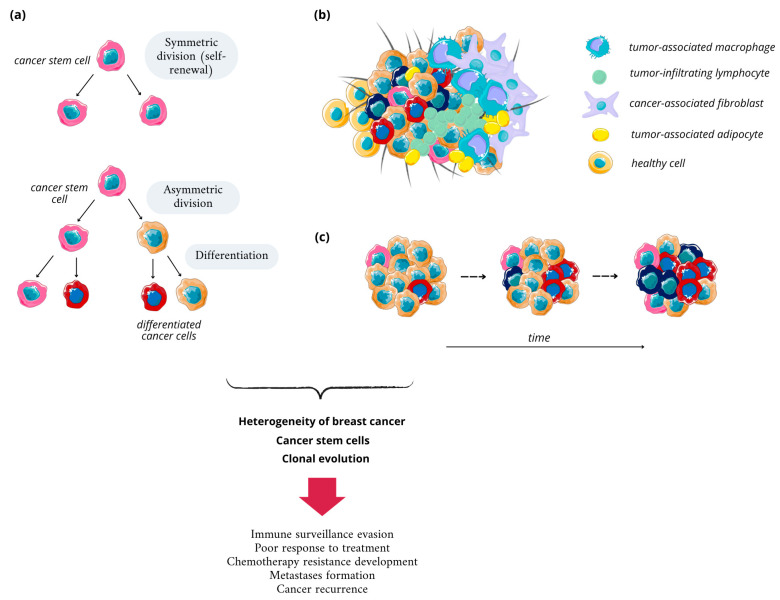
(**a**) Cancer stem cells (CSCs) undergo either symmetric division, generating two new stem cells, which is known as self-renewal, or asymmetric division, where one CSC and one non-stem cancer cell is formed. These non-stem cancer cells further undergo differentiation, giving rise to various populations. (**b**) A heterogeneous tumor microenvironment (TME) consists of a variety of cells, including tumor-associated macrophages (TAMs), tumor-infiltrating lymphocytes (TILs), cancer-associated fibroblasts (CAFs), tumor-associated adipocytes (TAAs), and different populations of cancerous cells. (**c**) Clonal evolution of cancer cells. All of these—heterogeneity, presence, features of cancer stem cells, and clonal evolution—contribute to cancer progression in a number of ways. Created with Servier Medical Art, on a license CC BY 4.0 (https://smart.servier.com/).

As mentioned above, breast cancer is a complex disease, characterized by different genomic, epigenetic, transcriptomic, and proteomic characteristics of cancer cells, as evident from the Cancer Genome Atlas Network analysis on over 800 BC cases published in 2012 [30]. The heterogeneity in BC, and cancers in general, reflects the successive growth of different cell subpopulations during carcinogenesis. According to the key concept in cancer biology, it originates from a single cell and then undergoes clonal evolution (Figure 2). Progression of BC starts from a normal epithelial cell and develops to metastatic disease in multiple steps, involving numerous metabolic conversions and the emergence of various cellular populations with altered characteristics. Some altered cell populations persist and gain dominance, while others reduce in number, creating a complex cellular landscape [31]. Throughout carcinogenesis, cells acquire genetic mutations or epigenetic alterations. These changes, such as the activation of growth pathways or inactivation of tumor suppressor pathways, provide growth advantages over neighboring cells [32].

A high degree of diversity is often observed within a tumor from the same patient, and this is known as intratumor heterogeneity. It is an important driver of cancer evolution, which through diversification allows cancer cells to endure various stresses, including selective pressure caused by chemotherapeutics. Also, heterogeneity plays a role in the metastatic activity of cancers. Consequently, it largely determines responses to therapy and further prognoses [2,33]. Genetic heterogeneity is represented by the presence of different cell subpopulations that have different mutational changes and/or copy number aberrations within their DNA. Epigenetic heterogeneity concerns the influence of external factors that affect the expression of certain genes, such as DNA methylation, chromatin accessibility, and nucleosome occupancy, while proteomic heterogeneity refers to the presence of different proteins, their levels, modifications, and signal transduction. The dynamics of transcriptional state, distinguishing different cell clusters, is also known to contribute to clinically relevant, functional intratumor heterogeneity [2,34]. Alterations on all of these levels are expressed as the phenotypic diversity of cancer cells, covering differences in cell morphology, proliferation rates, and angiogenic and metastatic activities, along with the presence of various surface markers [35].

Cancer cell heterogeneity relies on four main basal mechanisms. Firstly, the cell-of-origin differentiation state should be mentioned since numerous phenotypes of cancer cells can arise from the same origin cell, depending on the genetic event that initiates carcinogenesis. The above-described genetic evolution of cancer, when apart from the initiating, driver mutation in the origin cell, proliferating cells accumulate additional aberrations in their genetic material, is considered the second mechanism. Furthermore, the plasticity of stem cells, described as their ability to dynamically switch between cancerous and non-cancerous phenotypes, is a driver of cancer heterogeneity; cancer stem cells (CSCs) are able to self-renew and form more stem cells, differentiated cells, or tumor cells, while differentiated tumor cells can undergo dedifferentiation, enriching the tumor in more cell populations. Lastly, the tumor microenvironment (TME), consisting of fibroblasts, immune cells, endothelial cells, and blood vessels, is of equal importance (Figure 2). The presence of cytokines and growth factors, secreted by TME components, increases heterogeneity and reduces the sensitivity of tumor cells to growth inhibitors and therapeutics [36,37].

### 2.3. Breast Cancer Stem Cells

Breast cancer stem cells (BCSCs) were first identified in 2003; since then, they have been extensively studied. As indicated in the above paragraph, CSCs possess the unique ability to self-renew over the long terms, along with other distinctive features such as the capability to initiate tumor growth/regrowth and differentiate into non-stem cells. CSCs are characterized by metabolic reprogramming typical for cancer cells, and unlikely regular stem cells such as upregulated glycolysis, fatty acid oxidation, pentose phosphate pathway, and glutaminolysis satisfy the high energy demand of cancer cells in the expanding tumor [38]. Self-renewal can occur either in a symmetric cell division, which generates two daughter stem cells, or asymmetric division, where one CSC and one differentiated non-stem cell is formed (Figure 2) [39]. A number of cell types can give rise to CSCs, including normal adult stem cells, from which they are most likely to evolve due to the accumulation of mutations during their relatively long lifespan, progenitor cells, differentiated cells, fused cells, and bulk cancer cells. The transformation from progenitor and differentiated cells occurs mostly upon acquired mutations in genes encoding self-renewal traits of the cell, but this process requires the gathering of multiple mutations and, taking into account the shorter lifespan of such cells compared to stem cells, is rarer [40].

One important role in dictating the fate of stemness and differentiation is played by the microenvironmental features. Biomechanical signaling in the tumor microenvironment (TME), extracellular matrix stiffness, and shear stress regulate the expression of stem cell markers, activate the pathways responsible for the maintenance of CSCs, and influence cellular proliferation, migration, and invasion [38]. CSCs are associated with metastatic processes, as highlighted by an established link between their formation and epithelial-to-mesenchymal transition (EMT), a driver of cancer cell phenotypic change, loss of cell–cell adhesion, and, subsequently, dissemination [41]. While comparing the dysregulated signaling pathways stimulating stemness and self-renewal abilities in CSCs and cells undergoing EMT, an overlap can be easily noticed, hence the same pathways—e.g., Wnt, Notch, TGF-β, or PI3K/Akt—are involved [42]. CSCs through TGF-β, matrix metalloproteins (MMPs), and vascular endothelial growth factor (VEGF) release and promote EMT, participate in pro-metastatic extracellular matrix remodeling, and enhance vascular permeability, respectively [38].

CSCs also promote BC progression via creating an immunosuppressive environment to enhance immune evasion. The most notable mechanism is that CSCs can recruit macrophages and induce their polarization into M2 phenotype, which is considered tumor-promoting. Such tumor-associated macrophages upregulate stemness-inducing transcription factors and increase BCSC population. Moreover, BCSCs present enhanced abilities to escape immune surveillance through the overexpression of cytotoxic T-cell inhibitory molecule programmed death-ligand 1 (PD-L1), as well as inhibitory adaptive immune checkpoints [43,44].

### 2.4. Standard Diagnostic and Therapeutic Strategies

#### 2.4.1. Breast Cancer Diagnostic Methods

In clinical use, there are several breast cancer diagnostic modalities. Despite being introduced in the late 1970s, mammography is still one of the most commonly used diagnostic and screening methods in BC. After initial reports on the high sensitivity of this method (90–95%), it has become apparent that it is lower than it had been believed at the beginning [45]. The introduction of 3D tomosynthesis mammography brought considerable improvement in sensitivity, and therefore in cancer detection rates, reducing the risk of false positive diagnoses [46]. Ultrasonography is considered the basic diagnostic method, especially for young women, and is often used as a complementary examination to mammography. It is inexpensive and relatively easily available; however, it has a serious drawback—it may not detect all types of cancer due to their similarity to the natural glandular tissue of the breast [47,48]. Breast magnetic resonance imaging (MRI) is more sensitive and accurate than ultrasound and mammography, enabling the imaging of breast morphology and detection of pathological changes at early stages of the carcinogenesis process. Moreover, due to multiparametric assessment of breast lesions, it allows for discrimination between benign and malignant ones [49]. Apart from these imaging methods, breast cancer biomarker analysis and microscopic examination with immunohistochemical properties assessment of the tissue samples taken in biopsy are performed. Analysis of the biomarkers present in blood serum is not as established in BC diagnostics; the reason for this could be that, in early disease, the circulation levels of certain proteins that could serve as BC markers are below the detection limit of current analytical techniques [8]. To date, the only FDA-approved BC biomarkers are cancer antigens CA 15-3 and CA 27-29, along with HER2 and circulating tumor cells (CTCs). Quantification of CA 15-3 is performed as an immunochemical test with detection based on electrochemiluminescence; the technique used for CTC isolation is separation on immunobeads, capturing CTCs through their specific surface markers [50].

Needle aspirations and surgical biopsies are standard methods for diagnosing breast abnormalities. While excisional biopsies are occasionally necessary and reserved for cases where needle biopsies are technically unfeasible or unsafe, needle biopsies provide sufficient tissue for ancillary testing—core needle or fine needle aspiration is performed as the initial diagnostic step, allowing for planning the most suitable pre-operative treatment for patients requiring surgery due to malignant or high-risk lesions. Such preoperative diagnosis by core biopsy is obligatory due to its significance in precise surgical management and patient segregation [51,52]. The collected sample is further chemically treated, sliced, and subjected to histological examination. Tissue specimens can be formalin fixed and paraffin embedded to perform immunohistochemical staining, allowing one to detect and localize specific antigens; immunohistochemistry (IHC) is the main method used to distinguish between molecular BC subtypes [53].

However, there are concerns regarding cancer cell intravascular dissemination and cancer cell spread after the needle biopsy procedure, although no significant adverse impact on long-term survival or distant metastases development had been noticed [51]. Core biopsies of suspicious breast lesions can be challenging to perform in some locations [54]. Another important drawback is related to the fact that the collected sample contains only a fragment of the lesion, which does not necessarily comprehensively represent the tumor due to its high potential heterogeneity [55]. In addition, on small tissue specimens taken, it can be challenging to distinguish between some malignancies and benign conditions, and such interpretational difficulty can result in misdiagnoses with potential adverse outcomes for patients [56]. Also, a high percentage of errors can result from the low amount of cells in the sample, the presence of blood, or inflammatory infiltration [57].

Overall, the clinical and cost effectiveness of these diagnostic modalities appear to be insufficient. Taking into account variations in the resolution, sensitivity, specificity, and selectivity of current methods, as well as their limitations, developments are needed to satisfy the primary aims of BC diagnostics in terms of early detection, proper characterization, and the monitoring of tumor response to therapies [58].

#### 2.4.2. Standard Therapies in Breast Cancer

The majority of BC cases (>90%) are non-metastatic at the time of diagnosis. Therapeutic goals are tumor eradication from the breast and lymph nodes, as well as prevention of metastases and recurrence. In metastatic BCs, prolonging survival with a focus on restricting treatment-related toxicity is key [59,60]. In the treatment of all breast cancer subtypes, local (surgery, radiotherapy, photothermal, or photodynamic therapy) and systemic treatment methods, mainly chemotherapy, are in common use [61]. One of the main techniques to dispose of the tumor is surgery, which may be performed in early stages of carcinoma as well as when the disease is advanced. The type of conducted surgery, i.e., breast-conserving approach or mastectomy, depends on the severity of the BC, size, and exact location of the tumor [62]. Radiotherapy is used at all stages of BC treatment, from pre-invasive forms (as a permanent part of breast-sparing treatment) to the palliative irradiation of distant metastases. Irradiation used as a complement after surgery lowers the risk of relapse of the tumor and increases patients’ quality of life [63].

Systemic therapy is dependent on BC molecular subtype. In HR+ cancers, endocrine therapy, aimed at inhibiting estrogen-promoted tumor growth, is a primary choice, using aromatase inhibitors, selective ER modulators, and ER antagonists [60]. The treatment of HER2+ subtype is currently mainly conducted through monoclonal antibodies such as trastuzumab, tyrosine kinase inhibitors, and antibody-drug conjugates targeting the extracellular domain of this receptor. The following modulation of downstream signaling effectors dependent on HER2 signaling enables recruitment of immune cells to exert antibody-dependent cytotoxic effects, giving a favorable clinical outcome [64]. TNBC is an indication for complementary chemotherapy, when two- or three-drug anthracycline-based regimens are usually used in both preoperative (neoadjuvant) and post-operative (adjuvant) settings. Despite well-known short- and long-term risks, it remains an essential strategy in remission achievement and recurrence prevention [65]. Many neoadjuvant and adjuvant chemotherapy regimens can be considered. Apart from anthracyclines, such as doxorubicin or epirubicin, other drug classes can be used, such as taxanes (paclitaxel), antimetabolites (capecitabine, gemcitabine), and vinca alkaloids (vinorelbine) [66]. Immunotherapy is also applied, with good clinical outcomes, in HER2+ breast cancer and TNBC due to the high amount of tumor-infiltrating lymphocytes and high expression of programmed death ligand 1 (PD-L1), which is the main target of this treatment strategy [67]. The fields of immune-oncology and targeted therapies are constantly developing, but are still burdened by their high costs and substantial side effects [68].

Despite all of the advances, BC treatment regimens still face problems circumventing their efficacy, with resistance to therapies being the main issue. After initial positive response, cancer cells can eventually acquire resistance to basically all therapeutic agents, with epigenetic, proteomic, metabolic, and microenvironmental cues enabling some of them to survive under the selective pressure of anticancer treatments and initiating populations characterized by a higher degree of malignancy [69]. Some of the classical, well-established adaptations of cancer cells, enabling the survival of applied treatments, are an upregulation of drug efflux and detoxification systems, drug-metabolizing enzymes, and the activation of antiapoptotic pathways [70]. Apart from ABC transporters upregulated in numerous cancers (P-glycoprotein, multidrug resistant protein-1), breast cancer resistance proteins (BCRP/ABCG2), initially cloned from a multidrug-resistant BC cell line, play a crucial role [71].

The contribution of breast cancer stem cells to chemotherapy tolerance is also well studied. Since CSC asymmetric division can lead to the generation of multi-lineage differentiated cancer cells, it increases heterogeneity in BC and the development of populations of cells that are not sensitive to current therapies. In this manner, BCSC-induced heterogeneity promotes chemoresistance, with non-uniform response to therapy leading to further disease recurrences [44]. Additionally, CSCs present a low degree of differentiation, with a high ability to repair damage to genetic material, allowing maintenance of their ability to divide. Stem cells reside in specific niches, the components of which secrete factors stimulating BCSC self-renewal and support the metastatic activity of tumor cells. CSC niches are hypoxic and rich in reactive oxygen species (ROS); such an oxygen-low environment reduces the susceptibility of cancer cells to treatments, mainly radiotherapy, and therefore, being placed in such an environment allows them to develop resistance [72,73]. CSCs largely remain in dormancy, i.e., their cell cycle is arrested at the G0/G1 checkpoint; at this point, CSCs are inactive, evading cell cycle-affecting chemotherapy, and enter the next phase of the cell cycle under conditions that do not threaten their genetic material [73]. The characteristic for CSCs elevated expression of aldehyde dehydrogenase-1 (ALDH1), which catalyzes retinol oxidation at the early stages of stem cell differentiation, is an important factor in inducing chemotherapy tolerance [74]. Like bulk cancer cells, CSCs overexpress ATP-binding transporters, increasing drug efflux, leading to the reduction of intracellular drug accumulation below the therapeutic dose [75]. Some of the key oncogenic pathways activated in CSCs, including BCSCs, confer drug resistance, such as Wnt/β-catenin, NF-κB, Notch, Hedgehog, TGF-β, and Hippo [76]. BCSCs are reported as being implicated in the chemoresistance to drug classes such as anthracyclines, aromatase inhibitors, estrogen receptor antagonists, recombinant monoclonal anti-HER2 antibodies, and paclitaxel—all utilized in the treatment of BC [39,77].

**Figure 3:**
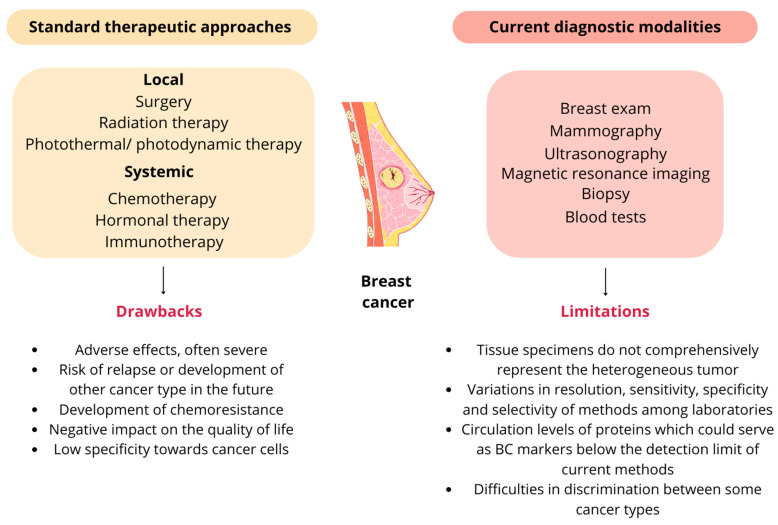
Summary of current treatment and diagnostic modalities in breast cancer with their drawbacks. Created with Servier Medical Art (https://smart.servier.com/).

The scheme presenting the current treatment and diagnostic modalities in BC with their drawbacks is shown in Figure 3.

## 3. Structure and Properties of Quantum Dots

### 3.1. Physicochemical Properties of QDs

The term “quantum dots”, coined by Brus et al. [78], is derived from the quantum confinement effect, which accounts for the difference in the electronic structure of bulk materials in comparison to semiconductor nanocrystals. In nanosized particles, the movement of excitons is constrained, while their energy remains basically unchanged. With the decrease of the particle size below the exciton Bohr radius, the excitation energy necessary to create electron-hole pairs increases [79]. The particle size is inversely correlated with the band gap, and likewise with the difference in energy between the valence band and the lowest conduction band. Therefore, the quantum confinement effect accounts for the unique electro-optical, photochemical, and magnetic properties of QDs, which can be altered by changing the particle size, composition, and structure [80].

**Figure 4:**
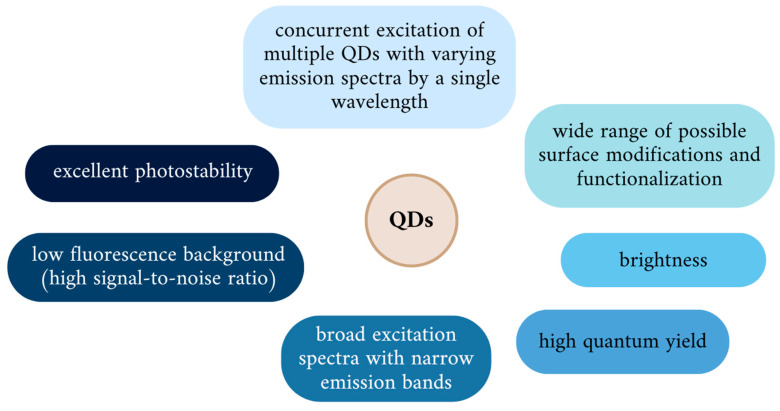
Features of quantum dots. Created with Servier Medical Art (https://smart.servier.com/).

By modifying QDs composition or regulating synthesis parameters, particles emitting the full spectrum of fluorescence, from UV to IR, can be obtained during a single process. Smaller QDs (2–3 nm) emit fluorescence at wavelengths corresponding to blue and green light, and along with the increase in core diameter up to 10 nm and changes in surface-to-volume ratio, their color of emission switches through yellow and orange towards red, characteristic for larger QDs. Hence, several probes constructed of QDs, varying in size, emitting fluorescence in different colors under UV irradiation, can be obtained using one type of semiconductor material [14,81]. This makes QDs ideally suited for multispectral imaging, due to the use of a single excitation wavelength to induce size-tunable color emission, as tools for multicolor detection and long-term imaging. A remarkable role in real-time tracking of single cells or molecules can be performed by QDs since they meet the requirements of rapid, continuous imaging of their targets through stable and strong bright fluorescence signals [82]. Moreover, they present an adjustable emission spectrum, large Stokes shifts, and slow decay [15,83]. Their superiority over conventional probes based on organic dyes and fluorescent proteins is manifested by excellent photostability, with a long fluorescence lifetime and resistance to photobleaching, exceptional brightness reported to be 10 to 20 times higher than other imaging probes, high signal-to-noise ratio, and low fluorescence background (Figure 4) [84]. Obviously, the specific properties and particle behavior are dictated by a certain combination of particular intrinsic and surface features, characteristic for individual QDs.

### 3.2. Types of QDs

Typical QDs are comprised of an internal core, covered by a shell composed of a semiconductor compound, which enhances their optoelectronic properties and stability along with toxicity reduction. The shell is usually composed of higher bandgap materials, e.g., ZnS, ZnSe, or CdS. Also, core–shell QDs are often provided with additional layer(s), serving as a surface area, which provide a high capacity of binding molecules of interest and can be further modified with synthetic or biological ligands such as proteins, antibodies, or oligonucleotides to target specific types of cells and intra- or extracellular structures [13,85]. Such ease of modification enables a broad range of applications, due to which QDs have earned their position in science. Conventional QDs are colloidal nanocrystals, the core of which consists of elements from the II–VI (e.g., CdTe, CdSe, HgS), III–V (InP, GaAs), or IV–VI (PbS, PbSe) groups of the periodic table. The II–VI QDs are well-established imaging agents in vitro due to their ease of preparation, enabling one to obtain multicolor, full-spectral QDs during a single procedure, while IV–VI QDs, due to their smaller energy gaps, emit fluorescence in the near-infrared and mid-infrared spectral region [86]. However, given that these QDs contain heavy metals in their core, their use in biomedicine in vivo is restricted and hampers their practical application due to presented toxicity towards living cells [87]. However, this issue is still discussed within the scientific community and is somehow controversial because of the limited insight into the toxicology of QDs. The results of cytotoxicity studies conducted on cell lines in vitro cannot be simply extrapolated to the complexity of a living organism, and at the same time acknowledged methods that would enable one to predict and analyze how specific QDs would behave in biological environments are lacking; however, there is a strong interest in resolving these safety issues, relying on the predictive approach [88,89].

**Figure 5:**
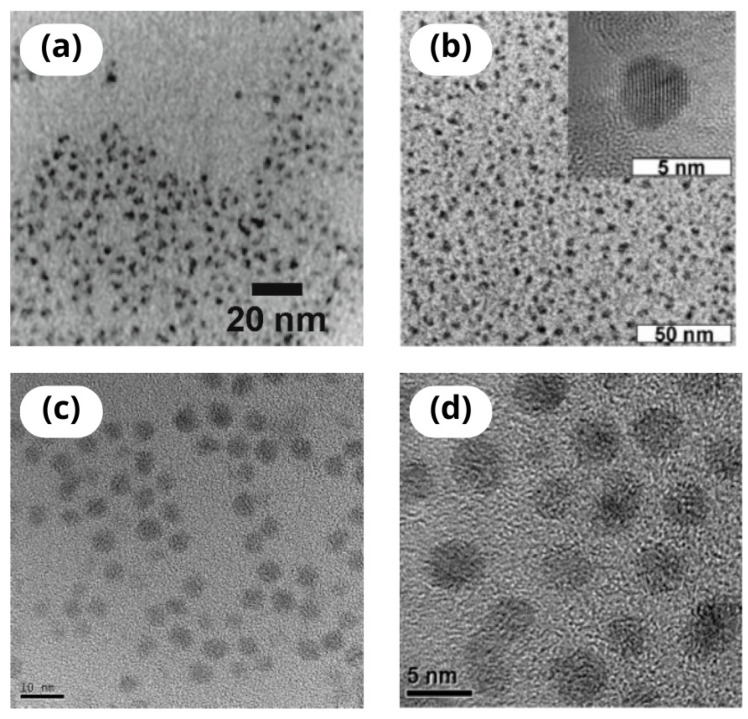
TEM images of (**a**) CdSe, (**b**) CdTe, (**c**) HgS, and (**d**) CQDs. Reproduced with permission from (**a**) Ref. [93], American Chemical Society; (**b**) Ref. [94], American Chemical Society; (**c**) Ref. [95], American Chemical Society; (**d**) Ref. [96] reproduced under the Creative Commons CC BY license.

Therefore, advancements regarding QDs have evolved either towards biocompatibility improvement via implementing various surface modifications, which shall be discussed further, or by inventing QDs made from materials other than heavy metal compounds. Consequently, silica (Si), black phosphorus (BP), and less known transition metal dichalcogenide QDs or MXene QDs (made up of two-dimensional inorganic compounds consisting of atomically thin layers of transition metal carbides or nitrides), have emerged [90]. Apart from binary semiconductor QDs, ternary I–III–V compounds, which include CuInS or AgInSe, are also used to develop QD-based probes. Although less toxic to cells since they are heavy-metal-free, they present low photoluminescence, low quantum yield, and poor stability to photo-irradiation [91]. The relatively new class of QDs and currently the strongest candidates to be used in biomedical applications are carbon-based QDs, divided into carbon (CQDs) and graphene (GQDs). The remarkable attention to this class of QDs stems from their features, such as lower or not-significant toxicity towards living cells, as well as environment, high quantum yield, better biocompatibility, and reduced production costs [92]. Exemplary transmission electron microscope images of different QDs are presented in Figure 5 [93,94,95,96].

It should be noted that, to be of any clinical usefulness, QDs must meet a number of requirements concerning their manufacturing and quality. For the needs of preclinical studies carried out by different research laboratories worldwide, QDs are synthesized on site, using various methods depending on expertise, resources, etc. Considering future introduction to the clinic, efficient, low-cost, and reproducible methods of QDs synthesis must be established. Also, the features of such QDs should ensure their scalability to the industrial level. Understandably, it is thought that QDs developed with the aim of in vivo applications shall not contain heavy metals [97]. The quality of QDs can be described as core crystalline perfection, the completeness of surface passivation, high dispersity, and size and shape uniformity. An additional feature is long-term material stability to ensure that it will not undergo uncontrolled decay in the biological environment [98]. These characteristics can be assessed with the use of the wide array of available techniques, e.g., transmission electron microscopy (TEM), X-ray diffraction (XRD), dynamic light scattering (DLS), and a variety of spectroscopic methods, including, e.g., emission and absorption spectroscopy or Fourier-transform infrared spectroscopy [99].

Regarding QD-based nanomedicine safety, the regulatory role of the Food and Drug Administration (FDA) and other agencies would be crucial from the early development stages, through clinical trials, to continuous market monitoring of products, if applicable [100]. Some recommendations point out that, before developing specific guidelines and principles upon resolving nano-toxicity issues, research on nanoformulations in the drug development area should be ceased [101].

### 3.3. In Vivo Issues: Uptake, Biodistribution, and Clearance of Quantum Dots

#### 3.3.1. Cellular Uptake of Quantum Dots

The successful utilization of QDs, like other nanoparticles, for biomedical purposes is dependent on the interactions between cellular components and introduced materials. To exert the specific effect in the cell, nanomaterials have to be taken up and internalized. The process of cellular uptake is dependent on several factors, among which the nanomaterial chemical nature is of most importance; further, particle size, shape, rigidity, surface charge, functional groups, and the resulting hydrophilic/hydrophobic character influence internalization and subsequent interaction with cellular components [102].

There are two major mechanisms in which cells can internalize external molecules: phagocytosis, which is an actin-dependent specialized uptake of large solid particles (≤0.5 μm), and pinocytosis, which is basically a fluid-phase uptake. The latter can be further divided according to the exact mechanism and involvement of certain proteins into macropinocytosis, caveolin-dependent, clathrin-dependent, and clathrin- and caveolin-independent endocytosis [103]. A general uptake pathway consists of three stages: endocytosis, sequestration in early endosomes, and translocation to late endosomes or lysosomes [104,105]. Experimental data gathered by various research teams indicate that endocytosis of QDs can occur in all the above listed manners, depending on their properties, as listed above; moreover, the main QD internalization route can comprise one or more endocytic pathways simultaneously [103]. Uptake via micropinocytosis is non-specific; therefore, it usually does not depend on exact QD properties, excluding surface functionalization, as specific molecules can stimulate such internalization. Several studies have confirmed that functionalization with PEG promotes the uptake of QDs by cancer cells and reduces non-specific internalization by healthy cells, since the presence of amine or carboxyl groups greatly enhances the uptake of such QDs in large amounts by various types of cell [105].

Clathrin-mediated endocytosis is the best known cellular uptake mechanism and is also considered the most important in the process of nanoparticle uptake [106]. This route has been shown to be a major one in the internalization of protein or peptide-QD conjugates, the same as for CdSe/ZnS QDs, although caveolae-mediated endocytosis and micropinocytosis were also shown to contribute to their uptake [107,108]. CdTe QDs coated with thioglycolic acid were determined to be taken up by ovarian cancer cells primarily through caveolae-dependent endocytosis [109]. Zhang and Monteiro-Rivere studied the uptake patterns of CdSe/ZnS QDs of 12 nm diameter coated with PEG, PEG-amine, and polyacrylic carboxylic acid. They observed that carboxylic-coated QDs were taken up in the greatest amount, localizing around the nuclear membrane periphery and in cytoplasmic vacuoles 24 h after treatment. Regarding the mechanisms of uptake, the authors have shown that these QDs in human epidermal keratinocytes were recognized by lipid rafts, but not by clathrin or caveolae [110]. Although mechanisms such as flotillin-assisted and fast-endophillin-mediated endocytosis, as representatives of clathrin- or caveolin-independent pathways, are also distinguished, they have not yet been studied with regard to QD uptake. This may result from difficulties in distinguishing these mechanisms from other ones, or lack of knowledge about the processes [106].

Carbohydrate functionalization of QDs has a different effect on their uptake, depending on the cell line used as a model and sugar attached to the particle surface. For instance, β-galactose CdTe/CdS QDs are efficiently internalized by asialoglycoprotein receptor-expressing liver cancer Hep2 cells, while α-glucose QDs show poor uptake. Silica QDs with D-mannose and L-alanine at their surface undergo rapid internalization by breast cancer MCF-7 cells [111]. Another study has shown the influence of galactose multivalency on the cell uptake mechanism and subcellular targeting. With the increase in galactose molecules per QD, the internalization pattern switched from lipid raft/caveolae-mediated endocytosis to clathrin-mediated endocytosis [112]. Uptake pathways also vary in the context of cell differentiation type and stage [105]. Importantly, QDs with an electric charge on their surface are either invisible or ae only shortly visible on the outside layer of cells. The distinctly positive or negative charge leads to their quick absorption by macrophages; therefore, after being injected intravenously, QDs are rapidly eradicated by the reticulo-endothelial system, consisting of liver, spleen, and bone marrow, preventing their accumulation in tumors [113]. Additionally, serum proteins adsorb to charged particles in an uncontrolled manner when introduced into an organism [114].

Similar findings concern carbon QDs since their primary internalization mechanism is found to be clathrin-mediated endocytosis, as shown in mouse kidney- and liver-derived primary cells [115]. As noticed by Yan et al., the uptake of carbon QDs by HeLa cells is dependent on their current cell cycle phase, with the influence of serum presence in culture media. In general, the highest uptake of both studied CQDs (PEI- and EPA-functionalized) occurred in the S phase of the cell cycle, and was lowest in the G0/G1 phase, which is consistent with previous studies [116]. Graphene QDs have been found to be internalized by gastric cancer MGC-803 and breast cancer MCF-7 cells, mainly through caveolae-mediated endocytosis [117]. For this type of QD, a correlation between size and uptake pattern was reported as smaller ones presented dynamin-independent and cholesterol-dependent pathways of internalization by dendritic cells, whereas larger ones have been taken up more slowly and only in the former mechanism [118].

In cell co-cultures, established by combining QD-labeled mesenchymal stem cells (MSCs) with the metastatic BC cell line MDA-MD-231 and primary BC cell line MCF7, the transfer of nanoparticles between stromal and cancer cells was studied by Saulite et al. to evaluate whether or not such nanoengineered MSCs could be used as drug delivery vehicles. According to their results, in serum-free conditions QDs excreted by MSCs were efficiently taken up by MCF7 cells via phagocytosis and clathrin/caveolae-dependent endocytosis, whereas the latter internalization pathway dominated in MDA-MB-231 cells, which was consistent with the internalization patterns of other types of NPs in these cell lines. Importantly, the transfer of QDs from MSCs to cancer cells was more efficient when they were co-cultured with the metastatic MDA-MB-231 line, compared to MCF7 primary cells. The QDs used in the study were CdSe/ZnS polymer-coated and carboxylate-functionalized [119].

Obviously, the intracellular delivery of QDs can be modulated in several ways, improving their biomedical applicability. Having knowledge of how the particular traits and modifications influence QD interactions with plasma membrane, which is the first barrier they encounter and, therefore, uptake by specific cells, including cancer cells, would enable greater control over this process. Selection of the delivery strategies would depend on the intended target cells or tissues and the desired QD activities. To facilitate the uptake and cytosolic delivery of QDs, specific peptides that increase endocytosis efficiency, the generation of protein scaffolds, polymer functionalization or small molecule attachment to the QD surface, and amino acid signal sequences can be made use of. Also, adjusting QD size, charge, shape, and other morphological and chemical features is of significance [120]. Moreover, the mechanical manipulation of cells can be conducted through physical methods like electroporation and microinjection [81]. However, it has to be considered that 2D cell culture studies, prevailing up to date, do not represent complex interactions in tissue environment and, therefore, more experiments on 3D cell or animal models are required.

#### 3.3.2. Distribution of QDs

To consider any in vivo biomedical application of QDs in humans, for both potential diagnostic and therapeutic purposes, the development of pharmacokinetic models to predict the effects of exposure to particular QDs over time is of paramount importance since their absorption, metabolism, and excretion patterns are expected to determine QD-related toxicity. Although different routes of xenobiotic administration exist, QDs are usually intended to be introduced into biological systems parenterally in an intravenous, intratumoral, or subcutaneous manner. The intravenous route is preferred over non-injectable approaches since particles are quickly distributed throughout the organism, enabling one to apply lower amounts of them. A specific route can be chosen to better suit particular applications, as in the case of the intratumoral injection of QD-anticancer drugs or radio/photosensitizer conjugate to accumulate at the tumor site when approachable. It is acknowledged that QDs after intravenous injection do not remain in circulation but tend to deposit in tissues and organs, sometimes exhibiting long-term accumulation [121]. The literature contains mentions on PEGylated CdSe/ZnS QDs, which have been observed to linger for up to 2 years in the mouse system [122]. Therefore, biodistribution-associated aspects shall be extensively studied on the cellular level as well as on tissues, organs, and whole organisms.

As can be expected, the subcellular distribution of QDs depends on a number of their physicochemical features. Regarding size and surface properties, it has been observed that smaller QDs functionalized with, e.g., cysteamine, apart from distributing in cytoplasm, are able to enter the nucleus, while non-functionalized larger QDs mainly accumulate in cytoplasm [123,124]. A classic example of core–shell QDs are CdSe/ZnS QDs, which, after uptake by HeLa cells, are mostly subjected to lysosomal distribution with a minority of particles locating in mitochondria, endoplasmic reticulum, and Golgi apparatus, to which QDs have been translocating from other organelles. Concerning their disposal, less than half of QDs underwent lysosomal exocytosis and endoplasmic reticulum/Golgi pathway, with approximately 60% of particles remaining internalized [107]. According to several studies, amine-functionalized cadmium-based QDs were found in the lysosomes in lower amounts than those with negatively charged carboxylic ligands. The same exocytosis pathways are reported for carbon-based QDs; distinctively, these nanoparticles often accumulate not only in the cytoplasm but also in the nucleus. What is surprising in light of their popularity in biomedical research, a scarce number of studies have investigated carbon-based QD trafficking patterns in mammalian systems at the cellular level [125].

The tissue and organ distribution of QDs is investigated primarily in mouse models. Not only features of nanomaterials but also factors such as blood flow, vascular permeability, plasma protein composition, and phagocytic and complement systems affect particle spatiotemporal spread [126]. Due to the direct contact between bloodstream and endothelial cells and capillaries in the liver, spleen, and bone marrow, the initial rapid distribution of NPs—including QDs—occurs in these organs [127]. Therefore, size seems to be of primary importance for their distribution and tissue accumulation; small-sized QDs are more suitable to cross endothelial cell monolayers, escape from the circulatory system, and enter target cels [123].

Understandably, colloidal QDs with a heavy metal core, unprotected with a surface polymer or ligand coating, more likely cause acute and chronic toxicity in tissues and organs where they accumulate, such as the liver, kidneys, spleen, lungs, or bones; this can be further reflected in blood profile abnormalities. In comparison to the liver and spleen, imaging experiments have revealed some patterns of surface coating-dependent distribution in major organs such as the liver, skin, bone marrow, and lymph nodes. Ligand-dependent distribution has also been documented [125]. However, the accumulation of QDs does not necessarily lead to their late toxicity; for instance, InP/ZnS QDs injected into mice, despite assembly, did not cause any detectable abnormalities for a long time period [128]. Nevertheless, such results should be taken with caution due to yet underexplored possible latent detrimental consequences. Some reports suggest that QDs may affect germline cells, which can be manifested, e.g., as prolonged oocyte maturation [129].

#### 3.3.3. Clearance of QDs

The hydrodynamic size of particles is crucial for developing diagnostic and therapeutic nanoagents. Owing to the average pore size of mammalian blood vessels, which is about 5 nm, particles sizes below this value equilibrate quickly between the bloodstream and extracellular space, while for larger particles this process occurs much more slowly. For instance, human IgG (10 nm diameter) takes about 24 h to equilibrate. Kidney filtration cut-off ranges between 6 and 8 nm pore sizes, making renal excretion highly dependent on nanoparticle size. QDs of diameter below this threshold could be rapidly cleared in this manner, while the larger ones would rather be subjected to uptake and further processing by the liver, among which some are excreted with feces or aggregate and are retained in the liver tissue [130]. Also, non-biodegradable NPs are primarily excreted by the liver [122]. However, these >8 nm particles, which do not undergo renal clearance, can accumulate and therefore interact with the system for longer time periods. As in previously mentioned processes, surface charge also plays a role—charged particles interact with biomolecules, which moreover limits their targeting ability, while neutral ones have fewer interactions with biological entities. Additionally, they may not be recognized by hepatic removal systems and readily pass through renal processing [123].

The importance of total body QDs clearance is not only related to potential toxicity associated with their long-time retention but also to the fact that metal-containing QDs deposited in organs can interfere with medical imaging methods like X-ray, MRI, ultrasound, and PET due to changes in imaging properties. It altogether emphasizes that analysis of body fluids, including urine and bile, should be included in human risk assessment after exposure to QDs [122]. Importantly, despite encouraging results concerning the suitability of CQDs for biomedical purposes, the understanding of their bioaccumulation, organ-specific effects, and overall impacts on organisms in real-world exposure scenarios, remains limited [131].

### 3.4. QDs-Associated Toxicity

In order to comprehensively and thoroughly address QD-related toxicity, one shaould identify which factors—and how—account for this effect in the case of particular QDs. Several patterns, however, have been formulated and are by now established, relying on the basis of numerous research study results. The material out of which a QD’s core is made can be intuitively stated to affect overall QD toxicity, yet is not the major factor which determines it [132].

CdTe and CdSe QDs seem to be the most explored type of QD. Cytotoxicity of this class has long been attributed to the release of Cd^2+^ ions from the core and their binding to key mitochondrial proteins resulting in cell apoptosis, but it is now known that another major contributor to cadmium-containing QDs’ detrimental effect on cells is ROS generation in a cellular response to exposure and lesser known estrogenic effects. The idea that it is not only the presence of Cd itself that is accountable for the toxicity of Cd-containing QDs was first reported by Su et al. [133], who observed that cytotoxicity caused by CdSe QDs to the K562 lymphoblastoid cells was more severe compared to CdCl_2_ solution, despite equal levels of Cd^2+^ internalized by the cells in both cases. It is suspected that irradiation with light during live cell imaging causes the photooxidation of QDs accumulated in cells, which further leads to electron transfer from QDs to O_2_ molecules, and in consequence the formation of superoxide anions, singlet oxygen, and hydrogen peroxide, disrupting the cellular redox homeostasis. Moreover, the unpaired electron holes that emerge as a result are able to react with the water molecules and generate hydroxyl radicals and other ROS entities [134], mediating oxidative damage to proteins, lipids, DNA, and cell structures, which may lead to cell death.

ROS formation as the additional toxicity mechanism seems to be universal and also accompanies CQDs and GQDs. Besides the electron transfer, the photo-induced energy transfer process to ground-state molecular O_2_ resulting in the formation of singlet oxygen can be mediated by CQDs [135]. GQDs, which share similar structural characteristics with CQDs, also exhibit a ROS-mediated mechanism, although their general level of cytotoxicity is relatively low [136]. Moreover, it has been noticed that GQDs, through their intrinsic peroxidase-like catalytic activity, are able to decompose H_2_O_2_ with the formation of hydroxyl radical [137]. GQDs have been found to activate both apoptosis and lysosome-based degradation, referred to as autophagy in macrophages, as a result of ROS generation [138]. According to current studies, it can be seen that, in general, low-concentration CQD solutions are not significantly harmful to human cells, but their cytotoxicity is observed to considerably increase as their concentration rises to a specific level, varying in dependence on other factors, such as conditions, the cell line used, etc. [139].

In the literature, proinflammatory effects mediated by QDs are also reported. CdSe/ZnS QDs have been demonstrated to induce the activity of proinflammatory cytokines in human primary monocytes, liver cells, and skin cells, representing the sites of exposure to QDs in parallel studies [140]. This observation raises the assumption that the proinflammatory effect may be related to QDs’ internalization patterns, which is highly dependent on the surface modifications. The internalization process involves numerous endocytic pathways, including receptor-mediated uptake or clathrin- or cell membrane lipid raft-dependent endocytosis [141].

Surface charge is another independent factor accounting for the toxicity of QDs and has been recognized by Nagy et al. [142] as prevailing over size and even surface functionalization. Indeed, multiple studies have confirmed that the positive charge of QDs’ outermost layer causes greater damage to cells than neutral ones or those with an anionic surface. This can be explained by the fact that the lipid bilaye- forming cell membrane has a net negative charge, hence cationic QDs are more likely attached to the cell surface and have a higher internalization rate. A high concentration of positively charged particles can lead to physiological damage of the cell membrane, an increase in proton pump activity, and alterations in intracellular ion flux, which overall leads to swelling of the cell and lysosomal rupture [143]. Most commonly, the functionalization of QDs with amine (-NH_2_) and carboxyl (-COOH) groups of positive and negative charge, respectively, was studied. Accordingly, Manshian et al. reported that COOH-CdSe QDs were, to a higher extent, internalized to cells in fibroblast cell culture and induced their cyto- and genotoxicity in a dose-dependent manner, in contrast to NH_2_-QDs of the same type, which had a minor effect on cell viability [144]. Other reports are consistent, e.g., assessment of cytotoxicity evoked by COOH- and NH_2_-functionalized ZnSe-coated CdSe QDs in the human lymphoblastoid cell line produced similar results [145]. Clearly, the overall particle charge is interrelated with the characteristics of its surface modifications. Significant differences in mouse fibroblasts viability after exposure to CQDs of positive (PEI-functionalized), neutral (PEG-functionalized), or negative (bare QDs) charge have been found. While PEI-CDs were able to enter the cell nuclei and cause cell cycle arrest in G0/G1 and G2/M phases, with an alteration in cell morphology, bare CQDs also promoted the proliferation of cells and cell cycle distortion. PEG-modified CQDs, on the other hand, have not induced any of these responses in the studied cell line [146]. Another study demonstrating differences in toxicity and uptake between differently functionalized GQDs has shown that the highest loss of viability among lung carcinoma A549 cells was caused by hydroxylated GQDs, while COOH-GQDs presented no cytotoxic effect in tested concentrations with no significant influence on autophagy induction in the tested cell culture. The authors speculated that the observed effects can be well explained by the differential activation patterns of MAPK signaling pathways, which varied in the case of each GQD, and in this case autophagy can be considered a response adapted by cells in order to survive [147].

The relationship between the size and toxicity of QDs is also well known. Generally, the bigger the QDs, the lower the viability and metabolic activity of cells treated with such, as established in numerous cell culture studies [13]. The positive size–toxicity relationship can be partly attributed to the reduced level of mobility within the cells and subsequent aggregation of particles [148]. However, it has to be highlighted that the cyto- and genotoxicity of QDs is also influenced by rarely considered factors such as level of QD aggregation, the presence of proteins in the biological milieu, exposure time, and surface area contact, since the toxic effects are not strictly correlated with the amount of internalized QDs. Hence, there is a need for the implementation of multiparametric analyses concerning particle toxicity [149,150].

It is known that the development of clinically useful QDs shall ensure mechanical, oxidative, and photolytic stability in physiological conditions [151]. Though the design of an optimal set of features should be specifically adjusted to the aim of QD use, in order to obtain therapeutically relevant QDs a balance in surface charges is required. Although cationic QDs could be preferred for gene or drug delivery purposes due to their promoted cellular uptake, finding the equilibrium between internalization effectiveness and sufficient toxicity reduction should be of higher importance [143]. Another crucial factor is stable coating. Encapsulating QDs in shells reduces their harmful effect, assuming the choice of proper coating material with regard to changes in distribution and pharmacokinetic properties of particles, depending on the intended application of the QDs. Although a ZnS shell is considered as a stable layer, effectively preventing the release of Cd^2+^ ions, the leaching occurs in acidic environment of lysosomes [152]. A more certain mean preventing QDs from degradation and therefore heavy metals from leaching is the addition of a secondary surface coating, like double-layered silica polymer, which has been shown to increase the resistance of CdSe/ZnS QDs to acids and therefore decrease their cytotoxicity [153]. Polymers as coating materials have a number of advantages, i.e., decrease of nanoparticle clearance rate, preventing them from recognition by the immune system. Moreover, the use of specific polymers such as hydrophilic, chemically inert, and neutrally charged PEG has a beneficial effect on reducing QD interaction with serum proteins and enhancing fluorescence emissions. The relevance in toxicity reduction and the assets of PEG in QD functionalization have been acknowledged in numerous studies [154,155]. The attachment of biomolecules, e.g., L-cysteine or glutathione, to the QD surface by now seems to be an effective approach to improve biocompatibility and reduce toxicity as well as lower the rate of undesired cellular interactions [156]. Also, the addition of transition metals, like nickel or manganese, to the outer layer represents a new approach to not allow ions to liberate from the QD core [157]. On the example of CdS QDs, it has been noticed that immobilization of these particles on the surface of clay nanotubes results in a reduced amount of released Cd^2^+ ions and a decrease in cytotoxicity [158]. Removal of oxygen-containing functional groups from the QD’s surface also seems to be useful due to the increased photostability of such QDs and lowered ROS generation rate [159]. The incorporation of QDs into liposomes or micelles has also been proposed as a method to reduce QD toxicity with retained surfaces and optical and electronic properties. However, despite numerous research studies devoted to both heavy metal-containing and innovative carbon-based QD functionalization and some success in this matter, challenges to the future clinical translation of QDs have not yet been resolved [151].

## 4. Quantum Dots in Breast Cancer Diagnostics

As mentioned previously, current diagnostic approaches include physical examination, laboratory blood tests, a combination of radiologic and nuclear magnetic-based noninvasive imaging modalities, and biopsy followed by histopathological property evaluation. Despite the wide range of available methods, each is prone to various errors. In light of rising BC prevalence and persisting high mortality from this disease, both technological and molecular advancements in BC diagnostics enabling precise, highly accurate, and unbiased identification of the exact type and stage of the cancer, facilitating early diagnosis and reducing the number of false-negative or false-positive results, are required. Therefore, a great effort is being put into the development of novel possibilities based on diagnostic probes or biomarkers to target specific molecular and genetic anomalies related to BC, which would offer high sensitivity, selectivity, and limited margin of error [58]. Nanomedicine, with an emphasis on QD-based approaches, can aid in meeting the goals of novel diagnostic techniques to allow selective and sensitive circulating or tissue biomarker quantification. Due to their unique optical properties, ability to be functionalized for targeted delivery, and versatility in both diagnostic and therapeutic applications, QDs seem to be highly valuable, useful tools. Their multifunctionality, combining imaging, sensing, and therapy, provides a comprehensive opportunity to cancer management, promising improved outcomes and reduced side effects.

### 4.1. Antibody Conjugated QD Nanoprobes

#### 4.1.1. HER2

The idea of detecting HER2 with the use of QD-based immunofluorescent nanoprobes is a known concept that emerged in the late 2000s. Since its expression is a basis to distinguish HER2-positive and -negative molecular BC subtypes, it is an important biomarker and attractive detection target [160].

Immunohistochemistry and fluorescence in situ hybridization (FISH) is currently among the most commonly used methods to assess HER2 status. IHC allows the detection of protein expression, while FISH detects genetic abnormalities associated with cancer. Unfortunately, IHC has its drawbacks, which include susceptibility to interfering factors, unstable sensitivity, high inter-laboratory variability, subjective interpretation, and a semi-quantitative nature with no standardized assessment. FISH, on the other hand, is complex, expensive, and labor- and time-consuming, and it requires specialized knowledge and equipment. Therefore, a quantitative, accurate, cost-effective and convenient method for detecting HER2 protein is desirable. QD-based probes have shown promising preclinical use for the detection of HER2 in BC, but they should be further investigated in a clinical setting [161]. The most specific antibody for the HER2 receptor is herceptin (trastuzumab, TZ), often used as a targeting molecule to localize HER2-expressing cells, and at the same time exerting a therapeutic effect. After microinjection into a blood vessel at the tumor site, QD/anti-TZ conjugate extravasate into the tumor microenvironment and bind to HER2 receptors at the cell surface, allowing cell imaging. Upon internalization, the complex releases free TZ, inducing apoptosis in cancer cells [160].

Rakovich et al. demonstrated that QD conjugation with a single variable domain of anti-HER2 antibodies can be successfully used for the immunolabeling of breast cancer cells and represents a potential biomarker that is more sensitive than conventional IHC [162]. Near-infrared QD bioconjugation with anti-HER2 antibodies with the use of EDC/NHS coupling methods performed by a different study group led to successful localization of both fixed and live tumor cells, as shown on the SK-BR-3 cells, which overexpress HER2 with MCF7 as a low-HER2 control [163]. QDs can also be used to determine HER2 expression level, as proposed by Miyashita et al., who developed a QD-trastuzumab-based immunohistochemical technique of single-particle imaging to quantitatively measure HER2 [164].

Apart from EDC/NHS coupling, streptavidin-biotin binding can be applied for conjugation purposes. For instance, it was used in the generation of QD-based sensors detecting HER2 and EGFR expression in the SK-BR-3 line during single-cell analysis. In a two-step protocol, these receptors were labeled with corresponding biotin-functionalized ligands and further captured by streptavidin-conjugated QDs with different fluorescence colors to enable simultaneous imaging [165]. Another QD-based double-color imaging was applied to evaluate the expression of HER2 and type IV collagen in tumor microenvironments, in the context of breast cancer invasive potential. This study was performed in fixed paraffin-embedded BC tissue samples instead of in live cells [166].

Nanoprobes consisting of QDs and single-domain antibodies (sdAbs) were implemented for the single- and double-photon detection and imaging of human micro-metastases, disseminated tumor cells in samples of metastatic BC mouse models, expressing HER2 or non-specific carcinoembryonic antigen (CEA), expressed by various cancer types. This allowed for the detection of HER2- and CEA-positive tumor cells infiltrating surrounding tissues or metastasizing to various organs, including the brain, testes, lungs, liver, and lymph nodes. The sdAb-HER2-QD and sdAb-CEA-QD nanoprobes, compared to conventional fluorescently labeled antibodies, provided a more accurate tool in detecting micrometastases in tissue sections, which is possible due to the lower photobleaching and higher brightness of the fluorescent signals. This leads to the conclusion that such nanobody-QD probes would help improve early cancer diagnosis and metastases assessment [167].

Since the extracellular domain of HER2 (HER2-ECD) can be cleaved by proteases and released into the bloodstream, its detection is also possible in serum samples. The method developed by Freitas et al., based on QDs as magnetic immunosensors, allowed for measuring concentrations of this analyte in serum. CdSe/ZnS QDs, conjugated to specific HER2-ECD antibodies through streptavidin-biotin chemistry, produced an electrochemical signal from the deposition of released Cd^2+^ ions in the acidic medium on the electrode surface while bound to the ligand [168].

Although the presence of ER and PR on the surface of cancer cells is grounds for a differentiation hormonal-positive (ER+/PR+) type of BC, to our best knowledge, there are no studies employing QD-based sensors in their detection and/or quantification.

#### 4.1.2. CA 15-3

Although carcinoma antigen 15-3 (CA 15-3) is a clinically acknowledged BC marker used to monitor response to treatment and disease recurrence, there are scarce reports concerning its detection by QDs. Cysteamine-CdS QDs, functionalized with anti-CA 15-3 antibodies, were used to study antigen–antibody interactions through spectroscopic and microscopic techniques. The developed fluorescent method allowed detection of the antigen of interest at very low concentrations (0.002 KU/L), which is less than the limits of detection offered by the most sensitive commercial ELISA kits [169]. The development of gold nanosphere-based immunosensors assembled on thiolated GQDs enabled the electrochemical detection of CA 15-3 in human plasma and MCF-7 cell lysates, with high selectivity and reproducibility. Given the linear proportion between the MCF-7 cell concentration and peak currents, the authors assume that this system could be useful in BC point-of-care diagnosis [170].

#### 4.1.3. Ki67

Ki67, as a marker of tumor cell proliferation, has been shown to have a significant impact on BC prognosis. The peak level of Ki67 occurs during the mitotic phase of the cell cycle, while in the G0 phase it is undetectable, allowing for differentiation between rapidly dividing and dormant cells. It is usually detected through the IHC technique. Yuan et al. developed a QDs-based multiple fluorescence in situ imaging method that stained nuclear Ki67 as a red signal and cytoplasmic cytokeratin (CK) as a green signal. This method allows simultaneous staining of nuclear Ki67 and cytoplasmic CK with a clear signal contrast, facilitating signal separation and quantification. This strategy was applied to examining tissue microarrays from 240 breast cancer patients, in which Ki67 and CK values and the Ki67/CK ratio were obtained for each patient, and their prognostic value for 5-year disease-free survival was assessed [171].

In another study, a QD-based two-color quantitative in situ imaging technique was used to investigate the co-expression of Ki67 (stained as a distinct red fluorescence in the nucleus of the cancer cell) and HER2 (stained as a bright green fluorescence on the cell membrane) and to determine the individual impact of these molecules on prognosis. The authors showed that the QD-based fluorescence imaging technique can help quantify Ki67 and HER2 co-expression in BC, and that Ki67 has a greater negative impact on BC prognosis than HER2 [172].

Water-soluble CuInS_2_/ZnS QDs conjugated to an anti-Ki-67 monoclonal antibody were also used to detect Ki-67 expression in BC. The QDs, which were hydrophobic and coated with octadecylamine, were encapsulated in an amphiphilic biocompatible polymer and then conjugated to anti-Ki-67 monoclonal antibodies to generate QD-Ki-67 bioprobes. These probes retained the optical properties of the undecorated QDs and showed no obvious toxic effects. In view of this, anti-Ki-67 monoclonal antibodies labeled with CuInS_2_/ZnS QDs represent a promising strategy for bioimaging and perhaps for use as a cell label [173].

### 4.2. QD–Aptamer Conjugates

Instead of antibodies, aptamers—functional single-stranded DNA or RNA oligomers—can be used for diagnostic purposes. Aptamers recognize and bind specific molecules, which can be cancer biomarkers, with high affinity, an prove to be less immunogenic and more stable over a range of different pHs, temperatures, and solvents, as an alternative to antibodies. Therefore, aptamer-based probes have gained rising attention as in vivo biosensing tools, mainly for cancer diagnosis, monitoring, and assessment of response to therapy [174].

Several studies have developed QD–aptamer sensors to detect and visualize breast cancer cells through specific biomolecules at their surface. The MCF-7 cell line is most commonly used as the BC model in such reports. Hua et al. performed the detection and collection of MCF-7 cells with the simultaneous use of two nanoprobes: nucleolin aptamer-conjugated CdTe QDs and mucin-1 aptamer-conjugated magnetic beads. Photoluminescence measurement allowed for the detection of up to 201 cells/mL, while square-wave voltammetric provided higher sensitivity, with a limit of detection equal to 85 cells/mL [175]. Mucin 1, as a transmembrane protein overexpressed, e.g., in BC, was targeted with a specific gold NP-based aptamer, also in combination with ATP aptamer-conjugated CdTe QDs. Due to electrostatic interactions and van der Waals forces between these two conjugates, a complex was formed, which after MUC1 recognition was internalized into MCF-7 cells and enabled the imaging of cancer cells [176].

In another approach, an electrochemical biosensor made of N-doped GQDs and phytohemagglutinin-L (PHA-L) deposited on electrodes was applied for the sensitive detection of MCF-7 cells. Strong bioaffinity and specificity of tetrameric PHA-L towards MCF-7 cells is based on the presence of mono-, oligosaccharides, and sialic acids on their surface. The sensor exhibited high biocompatibility, stability, enhanced current response, and extraordinary electrocatalytic performance in detecting MCF-7 since the limit of detection in human serum equaled 2 cells/mL, and 1 cell/mL when measured in PBS. The linear range of electrochemical MCF-7 detection was between 5 and 20 million cells per mL [177]. As reported by Borghei et al., a label-free system consisting of DNA aptamers conjugated to CdTe QDs can detect HER2 biomarkers in adhesive cells and cells suspended in complex environments (in serum samples), as well as in tumor samples, with a linear range of 15 to 1.5 million cells/mL [178]. It can be summarized that aptamer-QD sensors show promise for early BC detection and are worth further evaluation in both animal models and further clinical settings.

### 4.3. Non-Coding RNA Detection

Non-coding RNAs (ncRNAs) comprise a large group of RNA molecules, which are not subjected to translation into proteins, but are implicated in various cellular functions and, therefore, have biological importance. According to their size, ncRNAs are divided into long non-coding RNAs (lncRNAs), comprised of 200 or more nucleotides, and small ncRNAs, among which certain functional classes can be distinguished: microRNAs (miRNAs), small interfering RNAs (siRNAs), small nucleolar RNAs (snoRNAs), and other [179].

The most abundant among ncRNA types are miRNAs, usually built by up to 25 nucleotides and derived from transcribed hairpin loop structures. Along with siRNAs, they play key roles in modulating the expression of the genes that control a wide array of cellular and metabolic pathways. These two ncRNA types are structurally and biochemically similar—both are short RNA duplexes that induce gene silencing, but there are differences in how they perform this function. siRNAs are able to perform the cleavage of certain mRNAs, once they recognize its sequence and are bound through the activation of RNA-induced silencing complex (RISC) and endonuclease Argonaut 2 (AGO2). miRNAs also inhibit gene expression at the post-transcriptional level, but contrary they can target multiple mRNAs, while siRNAs, due to their total complementarity to certain mRNAs, have just one target. Conversely, multiple miRNAs can act in cooperation to regulate specific mRNAs [180,181]. The level and nature of the complementarity between the miRNA and target mRNA determines the gene silencing mechanism, as either cleavage and degradation of mRNA or AGO-independent translation inhibition [182].

Some ncRNAs have been found to act as tumor suppressors or oncogenesis drivers in multiple cancer types, participating in the regulation of multiple processes of cancer biology, including proliferation, apoptosis, metastasis, and angiogenesis. Therefore, various miRNAs are overexpressed in cancers. Regarding BC, multiple miRNAs characteristic for different subtypes have been identified. For instance, hormone-positive BCs overexpress predominantly miR-100 and miR-30 family members, along with miR-182 and miR-200. miR-21-5p and miR-125 have been connected with HER2+ subtypes, while TNBC has been found to be enriched in miR-10, 17, and 18, with the low expression of miR-200s, among others [183].

The conventional methods for miRNA detection include Northern blotting, oligonucleotide microarrays, and quantitative polymerase chain reaction (PCR), but all these require specialized equipment and are laborious and time-consuming. Among alternative methods, fluorescence sensing is being widely used to detect cancer-related miRNAs [184]. A number of reports describe the detection of BC-specific miRNAs, mainly miR-21 and miR-155, by QD-based probes. Specifically, 3D chitosan nanogel containing CQDs functionalized with complementary single-strand DNA (ssDNA), fabricated by Mohammadi et al., enabled the detection of miR-21 in MCF-7 cells with a particularly high sensitivity of 0.03 fM and linear range of 0.1 to 125 fM [185]. Recognition of miR-21 by MoS_2_ QD-based nanoprobes containing molecular beacons labeled with carboxyfluorescein, relying on the increase of green to blue fluorescence ratio resulting from the Förster resonance energy transfer (FRET) phenomenon, is also possible. The advantages of such a dual-channel system are the exclusion of false positive results and suitability to measure miR-21 levels in living cells, but the method covers the concentration range of 5–150 nM miR-21, which indicates rather low specificity [186]. In another approach, CdSe/ZnS QDs functionalized with DNA signal probes, cleaved by nucleases in the presence of target miRNA, are used in its fluorescence-based quantification. Such a duplex-specific nuclease-amplified (DSN) method enabled the detection of miR-148 and miR-21 in both cancerous (MCF-7, MDA-MB-231) and healthy cells, with a limit of 42fM for miR-148 with high selectivity—it did not detect mismatched sequences or other miRs [187].

**Figure 6:**
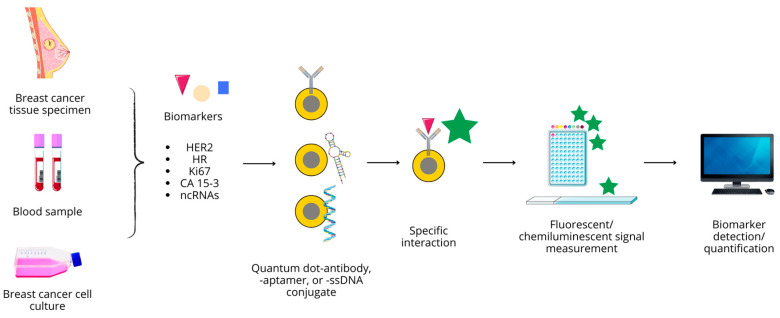
In a selective, specific detection of breast cancer biomarkers, such as those listed in this chapter, proteins and non-coding RNA, QD–antibodies, QD–aptamers, or QD–ssDNA systems are used. Specific interaction leads to signal generation, which is further detected and analyzed. To perform a successful detection or quantification, one component—a detected or detection molecule—is immobilized. Created with Servier Medical Art (https://smart.servier.com/).

As the detection of a single miRNA in disease diagnostics can produce high rates of false positive results, simultaneous detection of multiple miRs has been proposed to improve specificity. For example, antimonide QDs were used to construct an electrochemical biosensor for simultaneous quantitative determination of miR-21 and miR-155 in human serum samples, utilizing the strong interaction between antimonene (Sb) and single-strand RNAs due to a delocalized 5s/5p orbital, resulting in their high absorption. Within 80 min, both miRs can be detected in a range of concentrations between 0 and 1 pM [188]. A ternary electrochemical biosensor, consisting of gold nanoparticles/GQDs/graphene oxide functionalized with capture miRNA probes on carbon electrode, was utilized to detect three miRs: −21, −155, and −210, in serum samples. Detection limits of 0.04, 0.33, and 0.28 fM were determined, respectively [189]. miR-29a, reported to play a role in breast cancer EMT and invasiveness [190], has become a model for in silico molecular simulation of complementary DNA adsorption on GQDs and its binding with miR targets [191]. A simplified scheme of QD-based diagnostic processes is presented in Figure 6.

### 4.4. QD-Based Probes in TNBC Detection

Due to a lack of either hormone or HER2 receptors on the cell surface, TNBC is more difficult to detect at the molecular level than the other types. Heterogeneity among TNBC samples and their complexity hamper the assessment of reliable biomarkers. Overexpression of certain proteins can vary significantly among studies, as in the case of EGFR, reported to range between 13 and 78%. Another factor contributing to such variability is the lack of standardized IHC results analysis [192]. Although EGFR has not been accepted as a clinical TNBC biomarker, considering its high expression in TNBC it can serve as a molecular target for QD-based systems. Micellar formulations of InP/ZnS QDs conjugated with anti-EGFR were reported to accumulate in TNBC xenograft mouse models with no concomitant toxicity [193]. GQDs conjugated with a single-chain variable fragment of EGFR antibody located selectively EGFR-overexpressing cells of the MDA-MB-231 line. This complex was used as a double diagnostic-therapeutic (theranostic) agent loaded with cisplatin, released upon internalization by cancer cells [194]. Another study assessed the expression of EGFR along with collagen IV, the major TME constituent, by QD-based multiplex imaging and their quantitative determination in tissue samples. The obtained results allows one to distinguish between two groups of patients based on EGFR/collagen IV ratio, associated with 5-year disease-free survival, and negative correlation between EGFR and collagen IV amounts [195]. Besides EGFR, αvβ3 integrin is overexpressed in TNBC, and it also can be targeted [196]. Furthermore, QDs have been applied to detect some TNBC-related miRNAs, such as miR-155, miR-26a, and miR-221 [197].

The presence of α-folate receptors (α-FR) on TNBC cell surfaces enables the enhanced and more specific delivery of folic acid-functionalized probes to its cells. For example, N-doped GQDs with folic acid attached to their surface were confirmed as useful in the detection of BC cells, and the produced signal was linear in a range between 100 and 7.0 × 10^4^ cells/mL [198]. Quercetin-GQDs covered with folic acid presented a higher uptake rate by TNBC cells and greater accumulation, leading to higher apoptosis rate [199]. In addition, folic acid-functionalized CQDs as drug delivery systems were characterized by ease of crossing the endothelial monolayer and enhanced penetrability into tumor spheroids, used as a model for preclinical drug screening [200]. However, it must be kept in mind that the expression of α-FR is not specific for TNBC but is also observed in several other cancer types [194]. The findings discussed in this chapter are presented in Table 1.

### 4.5. QD-Based Multiplexed BC Biomarker Imaging

We have mentioned previously that the behavior of cancers, obviously including BCs, is highly dependent on the tumor microenvironment in which they are set. Investigating specific features of TME, characteristic for individual BC cases, would provide unique information that might aid in developing the personalized treatment strategies most suitable for each case. Although imaging methods currently in clinical use are not capable of producing multi-dimensional data from both cancer cells and their TME, it seems to be an ideal application for QDs [201]. With their use, the simultaneous detection of multiple protein biomarkers is possible in a single imaging session, as the detection of different targets is possible upon excitation of several probes by a single wavelength [202]. Such multiplexed imaging, enabling evaluation of functional, spatial, or temporal correlations between biomolecules, enhances the comprehensive profiling of breast cancer cells, aiding in the deeper understanding of tumor heterogeneity and biology.

Several modifications of the conventional immunostaining have been proposed to develop a multiplexed IHC procedure for the detection of numerous biomarkers; however, not only does it prolong an already several-days-long procedure but also has repetitive stages of demasking, blocking, staining, destaining, etc., which lead to biomarker loss and tissue deformation [203]. In fluorescent QD-based multiplexed imaging, an ideal molecular recognition system based on antibody-antigen specific interaction is mostly used. The labeling procedure can be a direct staining, when each of the primary antibodies is conjugated with one color of QD and used to stain tissue specimen in a single step, or indirect staining using a QD-secondary antibody complex to recognize a primary antibody bound to its antigen. Currently, the latter approach is more common in ex vivo tissue specimens and cultured cell examination [204].

One early study performed the imaging of HER2 and ER expression, together with their quantification and histomorphological observation of the cells in BC tissue sections [205]. In 2013, CdSe QDs were used to visualize Tn antigen in the MDA-MB-231 cell line and tissue microarrays made of cancerous and benign patient breast tissue samples. The Tn antigen, a truncated O-linked core glycan linked to the serine or threonine of mucin 1, is expressed in epithelial cancer cells, but not in normal cells. It enabled distinguishing between healthy tissue and benign and malignant cancers with 95% sensitivity and 90% specificity [206]. Five proteins, ER, PR, mTOR, EGFR, and HER2, were detected by QD-antibody systems in BC tissue specimens by Yezhelyev et al. [207]. In a more recent study, multiplexed imaging of ER, PR, HER2, and nucleoside diphosphate kinase 1 (NM23) was performed on formalin-fixed paraffin-embedded BC tissues, detecting their co-expression and spatial interdependence [208].

## 5. Advances in QD-Based In Vivo Imaging in Breast Cancer Studies

Given the previously discussed optical properties of QDs, BC researchers also aim to utilize them as in vivo tissue imaging probes in order to meet personalized oncology principles in the future. Indeed, there are reports of QDs being used in vivo in imaging BC xenografts, visualizing lymph nodes, and detecting metastases [201].

### 5.1. BC Xenografts Imaging

The visualization of grafted tumors in vivo is also possible through QDs, and several studies have demonstrated their successful use in mouse models. Also for this purpose, conjugating QDs with HER2 antibody to create a fluorescent immunoprobe targeting xenografts is a common practice [197]. For instance, a recent study reports the synthesis and use of hybrid CdSe/ZnS QDs-HER2 antibody nanoprobes in immunofluorescent BC xenograft imaging on a nude mice model. Upon intravenous injection, such probes have not displayed any obvious toxicity, and their high tumor targeting ability was an evident asset [209]. Besides HER2-Abs, other targeting molecules have been used. Liu et al. developed a CuInSe_2_/ZnS QD-CGKRK peptide conjugate, targeting orthotopic MCF10CA1a xenograft tumors in live mice. The probe had high photostability and bright luminescence and presented strong tumor specificity [210]. 4T1 breast tumor xenografts were successfully imaged by Ag_2_S QDs, but due to the lack of any specific surface functionalization, only passive tumor targeting through the enhanced permeability and retention (EPR) effect was possible; approximately 10% of the injected dose had been internalized by cancer cells [211].

### 5.2. Mapping of Lymphatic Nodes

Since the axillary lymphatic system (ALS) is the primary route for lymphatic drainage and the main pathway for lymphatic metastasis of BC, the preoperative and intraoperative detection and imaging of the ALS are critical steps in predicting lymphatic metastasis, planning surgery, and assessing disease prognosis. Mapping ALS through QDs is now widely studied in preclinical models as an alternative to LN dissection. In recent years, there has been a global increase in axillary lymph node (ALN)-negative BC cases, leading to growing interest in the sentinel lymph node (SLN), which is the initial and crucial site for lymph node metastasis in BC [197].

An undoubted benefit of using quantum dot (QD)-based imaging is its ability to simultaneously assess and differentiate lymphatic drainage from multiple distinct areas, a task that traditional lymphangiography methods like X-ray, MRI, or radionuclide imaging find challenging. Moreover, strong and lasting fluorescence facilitates easier detection and subsequent procedures like biopsy and dissection of the SLN [201]. Near infrared (NIR) fluorescence offers distinct advantages for imaging the lymphatic system compared to visible light, primarily due to its superior ability to penetrate the skin and subcutaneous tissues. NIR QDs, with emission wavelengths between 700 and 2500 nm, are particularly well-suited for mapping the lymphatic system, hence their tissue penetration depth exceeds 5 mm, allowing them to effectively image lymphatic structures, even in large animals. Additionally, in comparison to traditional fluorescent dyes, while using QDs the interference from tissue autofluorescence is minimal and facilitates deep tissue imaging [212]. Altogether, QD-based probes could be used instead of indocyanine green in performing imaging-guided surgeries.

In mice, Hama et al. has simultaneously mapped two lymphatic flows from the breast and the upper extremity with two NIR-QDs [213], while later on Kosaka et al. used five QDs with different emission colors injected at different sites to simultaneously image neck LN, thoracic LN, and ALN [214]. Another study confirmed the utility of CuInS_2_/ZnS QDs as tools in non-invasive SLN visualization in BC-bearing mice; in this research, In-based NIR-QDs were used [215]. Also, red fluorescent AlS/ZnS QDs have been shown to serve as a high-performance imaging agent for tumor drainage lymph nodes in vivo [216]. In a different approach, a bioluminescence resonance energy transfer (BRET) between QDs and nanoluciferase was utilized to achieve a self-illuminating system, enabling the production of lymph node images of high contrast with only small quantities of the imaging agent [217]. Another research team managed to image SNL in an orthotopic 4T1 breast cancer mouse model using ultrabright PbS/CdS QDs [218].

### 5.3. Detection of Metastases and Micrometastases

Difficulty in the early detection of cancer metastases is a known clinical concern with current imaging techniques that are able to detect only an already grown secondary tumor. Moreover, tumor outgrowth in a distant site is long preceded by early stages of metastatic spread, known as micrometastases. These are defined as cancer cell clusters of 0.2–2.0 mm diameter, emerging as an important factor in BC progression. Therefore, their rapid and sensitive detection is crucial. QD-based fluorescence, high-resolution multicolor imaging offers a potential solution; however, developments in this field are scarce [197]. To follow the metastasis process, Gonda et al. performed QD-mediated analysis of cell membrane protein dynamics, which is of key importance for cancer dissemination. They used QDs conjugated with antibody against protease-activated receptor 1 (PAR1), a pro-metastatic factor located in the cell membrane, to record the movements of cancer cells and PAR1 during metastasis in BC-grafted nude mice [219]. A double-staining method, based on commercially available red fluorescent QDs, outperformed the classical hematoxylin-eosin rapid staining in an intraoperative diagnosis of BC lymph node micrometastasis [220]. A combination of MRI and optical imaging allowed the visualization of ZnO NP/QD-labeled MDA-MB-468 cancer cell movement through mouse ALN upon grafting, with particularly high spatial resolution [221].

## 6. Potential of Quantum Dots as a Therapeutic Modality in Breast Cancer

### 6.1. Phototherapy

Phototherapy, which can be divided into photodynamic therapy (PDT) and photothermal therapy (PTT), is considered a non-invasive treatment modality in cancers, including BC. Both techniques are advantageous on multiple levels. Their use contributes to overcoming chemoresistance and cancer cell compensatory mechanisms, thus increasing the efficacy of therapy. Also, PDT and PTT affect tumor perfusion, as well as extracellular matrix (ECM) permeability, and such effects provide the opportunity to improve targeted drug delivery to cancer cells [222,223].

The treatment of cancers with the use of PDT techniques is based on irradiating tumors by a laser with a particular wavelength, which activates photosensitizer (PS) drugs that have selectively accumulated within the cancerous tissue (photosensitizer is removed from healthy cells in a few hours, while its elimination from cancer cells is constrained due to the poor development of lymphatic vessels) [224,225]. As a result of irradiation, a photochemical reaction is triggered, during which molecules of the PS transfer energy to the neighboring oxygen particles, generating singlet oxygen (^1^O_2_) and other ROS. These entities, through oxidizing lipids, proteins, and nucleic acids, disrupt cell function and induce cytotoxicity, leading to cell death [222,226]. Since ROS are characterized by a very short diffusion distance, they are able to react with macromolecules only in the nanometer range of the site where they have been generated. It also implicates locating PSs in close proximity to the target structure [219]. Given that tissue-depth penetration is limited to a maximum of 10 mm, cancer cell necrosis via PDT can be induced only in superficially located tumors. However, PDT can also be used complementary to surgery and chemotherapy to aid in the removal of larger tumors’ residues. The most significant advantage of PDT is that, due to restricted penetration of light, it does not induce phototoxic damage in healthy tissue, but only within the tumor [225].

PTT also provides low toxicity and invasiveness, but its spatiotemporal selectivity is not as high as in the case of PDT. In this approach, similar to PDT, photothermal agents after near-infrared (NIR) light irradiation generate local temperature increases in the mechanism of energy transfer through electron–phonon and electron–electron relaxation. PTT uses the relatively high power of light in order to achieve either subcoagulative (range 45–55 °C) or coagulative (55–100 °C) temperatures, the application of which leads to rapid cell death due to denaturation of vital proteins and cell membrane disruption, followed by secondary necrosis [227]. However, since there are no heat confinement mechanisms, a temperature gradient created by PTT spreads beyond the photocoagulation zone, inducing thermal damage to some non-tumorigenic cells [228]. PTT can target both primary tumors and early metastases, inducing apoptosis and autophagy and disrupting cellular signaling in breast cancer cells, as reported by Kadkhoda et al. [229]. Enhanced therapeutic effects in BC can be achieved through a combination of PTT, chemotherapy, and/or PDT—for example, hyperthermia induced by PTT was shown to sensitize cancer cells to PDT, and conversely, PDT increases their sensitivity to heat [230].

A number of studies have reported that combining photosensitizers with nanoparticles (NPs) results in improved PDT efficacy and a reduction of its adverse effects in the treatment of various cancers, including breast cancer. To be used as PS enhancers, nanomaterials should adhere to several requirements: (i) consistent emission spectrum of NPs to absorption spectrum of the PS in order to efficiently activate PS, (ii) high luminescence efficiency, (iii) ease of conjugation to the PS, (iv) non-toxicity, (v) water-solubility, and (vi) high stability in the biological environment [198]. According to some research, QDs exhibit the potential to function as efficient PSs themselves or energy donors for other PSs in PDT [231]. The limitation of the majority of widely used PSs is their low quantum yield in comparison to fluorescent dyes, which reduces their potential as theranostic agents for simultaneous fluorescence detection and PDT. In this regard, QDs can be used to enhance both these processes, activating PS either in an indirect excitation pathway (fluorescence resonance energy transfer (FRET)) or directly via triplet energy transfer mechanisms [232]. Owing to the functionalization-prone surface of QDs, photosensitizers can be attached to a QD and form a conjugate, where QDs facilitate the excitation of the PS and subsequent ROS generation. The broad absorption spectra of QDs allow using variable excitation wavelengths in order to activate particular PSs; in addition, the spectral properties of QDs can be easily modified to match the absorption spectrum of any PS by altering QD size or composition [233]. Apart from activating PSs via FRET, PS-QD conjugates are also capable of direct ROS generation, as shown on the HeLa cell line after using aza-BODIPY-CdSe QDs. Upon irradiation at 635 nm, enhanced phototoxicity and preferential localization of the complexes in cancer cells were observed [234].

Taking into account the issue of metallic QDs’ toxicity, CQDs and GQDs hold the greatest promise regarding their application in breast cancer PTT/PDT. Surprisingly, the concept of QD-based PDT seems to be underexplored—studies at clinical levels concerning the application of QDs in PDT of BC are lacking. Nevertheless, cell culture and animal model studies are being conducted [235]. Recently, the applicability of biocompatible tea polyphenol-derived CQDs in PDT was assayed in vivo on animal models. The study confirmed a high level of hydroxyl radical generation with concurrent minimal toxicity in healthy tissues after mild irradiation of mouse xenografts created by injecting 4T1 cells, representing stage IV human breast cancer, with an LED light. As a result of CQDs injection and light exposure, the tumors significantly decreased. Moreover, it was shown to have not damaged any major organs and did not produce any lesions, demonstrating that such QDs should be further studied due to their potential in anticancer PDT [236]. Ge et al. developed a GQD-based photosensitizer, able to generate large amounts of ^1^O_2_ in a multistate sensitization process, enabling them to obtain the highest quantum yield reported for PDT agents (~1.3) [237]. Female mice with subcutaneous breast cancer xenografts were subjected to GQDs injection followed by irradiation with white light, which resulted in tumor decomposition and no regrowth during the observational period of 50 days. In contrast, the tumors of mice in the control group were constantly growing. According to the authors, no noticeable toxicity associated with GQDs injection occurred. A successful application of N-doped graphene quantum dots (QDs)/titanium dioxide nanocomposites (N-GQDs/TiO_2_ NCs), photoactivated with NIR light, in reducing MDA-MB-231 breast cancer cells viability, was also reported [238].

Some studies show that QDs can serve as both nanocarrier and photosensitizer in PDT. The unique CQD clathrate-like structures conjugated with folic acid were synthesized and evaluated by Pandey et al. [239] in order to deliver metothrexate (MTX) to breast cancer cells, additionally serving as a PS in the following PDT. They have proved successful targeted delivery of MTX in high concentration selectively into malignant tissue, and subsequent irradiation of the conjugate with a near-infrared laser caused drug release and rapid ROS generation, improving the cytotoxic effect on cancer cells. This study confirms the favorable properties of CQD-MTX-FA conjugates, such as photo-responsiveness, long term photostability, high drug loading capacity, and biocompatibility. The pharmacokinetic properties of this complex were also beneficial, like decreased MTX clearance rate from the plasma. Thus, CQD-based structures seem to serve as an efficient drug carrier and are easy to functionalize to gain the ability of selective accumulation in tumor tissue and be used in synergistic photo-chemotherapy.

Another less toxic alternative than carbon/graphene QDs are InP/ZnS QDs, which have been used by Charron et al. to construct hybrids with chlorin e6 as the photosensitizer [240]. The authors reported that, despite successful coupling, there were no significant differences in TNBC, representing MDA-MB-231 cells’ viability after application and irradiation of the conjugate versus free PS, indicating the need of improvement of the hybrid design to enhance the energy transfer rate between QD and PS.

On the other hand, ZnO QDs may be used as PSs and therefore be applied in PDT. Irradiation with blue light (470 nm) led to higher cytotoxicity towards the MDA-MB-231 cell line than in the case of QDs only. This combination has been reported to increase lipid peroxidation rates, lactate dehydrogenase (LDH) leakage, apoptotic genes, and caspases 3 and 9 expression, suggesting this synergistic anticancer effect might be adopted in BC therapy. Also, a reduction in antioxidative enzyme catalase activity was noted [241]. ZnO QDs are acknowledged to exert an antiproliferative and cytotoxic effect on MCF-7 and MDA-MB-231 human BC cells. An additional advantage of their application as PS in PDT is a high degree of selectivity towards cancer cells [242], which meets the requirement that toxicity shall be induced only in a restricted area of malignant tissue, not damaging the neighboring healthy cells [243].

QDs can also be used in the development of dual PDT/PTT nanoparticles, integrating in a single particle two or more types of material: one acts as a photosensitizer, while the other presents high photothermal conversion efficiency. Upon combining a PS with QD, the nanoparticle surface can be functionalized in order to improve pharmacokinetic properties or ensure proper targeting [223].

### 6.2. Targeted Drug Delivery by QDs

#### 6.2.1. Targeting Cells of Destination

Implementation of QDs as drug delivery systems is presumably the most distinctive objective of current QD-focused biomedical studies due to the unprecedented opportunity to improve drug loading, targeting, and efficacy in anticancer treatments. Properly developed targeted drug delivery through QDs contributes to lowering their systemic toxicity and accumulation at the tumor site, via enhanced permeability and retention effect; it also provides an opportunity to bypass biological barriers, such as the blood–brain barrier [244]. It has already been proven on examples of liposomes, polymeric, or superparamagnetic NPs as drug carriers that such solutions improve the efficacy of treatments in comparison to free therapeutic agents [245].

To perform efficient drug delivery, QDs must be endowed with moieties, enabling them to target certain cells, e.g., cancer cells or subcellular compartments. Therefore, the attachment of biofunctional units to the QD surface, specifically interacting with recognition molecules, must be performed. In the case of directing NPs towards cancer cells, specific cancer-associated receptors, which they express/overexpress, are targeted to enable the selective uptake of QD–drug conjugate. Owing to the overexpression of receptors for folic acid (FA), hyaluronic acid (HA), transferrin (Tf), and biotin on cancer cells, these entities are most often used to decorate tumor-targeting QDs. Upon binding to the corresponding receptors and the following interaction, drug-loaded FA/HA/Tf-QDs can be internalized by receptor-mediated endocytosis and accumulate in cancer cells. In this uptake route, the QD–drug conjugate was shown to be not recognized by P-glycoprotein, the major resistance protein performing active drug efflux to the outside of cancer cells [246,247]. Tumor angiogenic cells can be targeted with an antibody against the VEGF receptor (VEGFR) [240]. The idea of encapsulating QDs within cancer cell membranes to be actively taken up by tumor cells was also reported. QDs delivered in such a way presented efficient tumor targeting, high aggregation within cancer cells, and good retention at the tumor site, showing an enhancement of PTT/immunotherapy outcome in mice [248]. In studies concerning targeting and eradicating breast cancer cells in vitro, all three coating biomolecules (FA/HA/Tf) were used.

The second part of creating a functional QD-based delivery vehicle is the stable attachment of the drug molecule, which requires the presence of a suitable functional group on its surface. In approaches using covalent conjugation, attachment through free amine or the carboxyl group of the drug molecule is most common. It enables a high drug loading rate but is also associated with its relatively slow release [217]. Direct adsorption and electrostatic interactions are not favored due to their low spatiotemporal specificity. It can be, to some extent, controlled with the use of coupling agents, but their application also requires the presence of free amine/carboxyl groups [249,250].

#### 6.2.2. Targeted Release

To ensure the functionality of nanocarrier-drug conjugates, the targeted release of the therapeutic cargo inside destination cells is crucial. Considering anticancer therapy, QD–drug complexes must remain stable throughout their journey towards the tumor and uptake by cancer cells. Only after accumulation in the cytoplasm will drug release be triggered. Moreover, such a release must rely on noninvasive methods, and the desired modality would be a possibility to monitor this process in real time [251].

The release-triggering stimuli can be divided into internal and external ones. Internal stimuli are related to specific conditions in the cancer cell or tumor microenvironment, such as lower pH in comparison to its physiological value in healthy cells and the bloodstream, which is a feature commonly made use of while designing controlled release systems. This difference stems from the altered metabolism of cancer cells, which turn to glycolysis instead of oxidative phosphorylation, leading to intensified lactic acid production [252,253]. For example, in acidic medium, amide bonds can be hydrolyzed; therefore, linking chemotherapeutic to QD with this type of connection enables pH-triggered drug release, as shown in the example of GQDs and cytarabine [254]. Another difference is a discrepancy in glutathione (GSH) levels, heightened in cancer cells due to increased exposure to ROS. To utilize this fact, the drug can be linked to the carrier through disulfide bonds, creating a redox-susceptible system since this linkage can be easily cleaved by GSH. Nanocarriers, including QDs, can also be made vulnerable to enzymes of increased activity in cancer cells, like proteases including cathepsins or hydrolases. Moreover, cancer cells, due to their rapid proliferation and accelerated metabolism, are characterized by increased ATP concentrations; therefore, ATP-responsive mechanisms are also developed [249].

**Figure 7:**
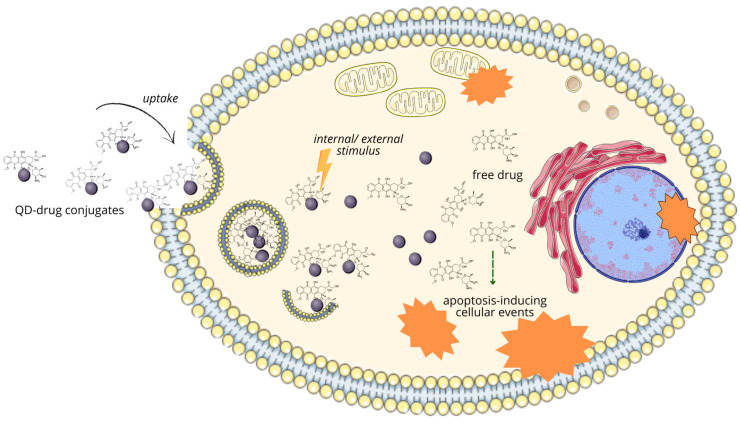
Schematic representation of chemotherapeutic release from QD–drug conjugates. Upon internalization, which may occur through macropinocytosis, caveolin-dependent, clathrin-dependent, or clathrin-/caveolin-independent endocytosis, an internal or external stimulus (e.g., pH, enzyme, redox conditions, ultrasound, magnetic field) triggers drug release from the complex. The free drug, according to its mechanism of action, causes different events, leading to cancer cell death. Created with Servier Medical Art (https://smart.servier.com/).

Among external stimuli, ultrasound, magnetic fields, temperature, and light can be listed. Thermo-responsive conjugates release the drug under conditions of mild hyperthermia (41–42 °C) induced by external sources. This feature is commonly used in photothermal therapy. Another concept is based on developing complexes that destabilize upon irradiation with light of a specific wavelength, as well as upon exposure to ultrasonic waves. Magnetic field-controlled drug delivery is a common approach to nanoparticle-based systems, including QDs. Currently, dual- or multi-responsive nanocarriers (e.g., pH/redox, pH/enzyme, etc.) seem to have gained the greatest attention [249,251,255]. The schematic representation of chemotherapeutic release from a QD–drug conjugate is shown in Figure 7.

#### 6.2.3. QD-Based Targeted Drug Delivery in Breast Cancer

In terms of potential clinical applicability, the highest expectations are placed on CQDs as a nontoxic, biocompatible nanomaterial, with good water dispersity and high drug loading capacity. Non-carbonaceous QDs are scarcely studied in relation to targeted drug delivery in BC cellular models. In a recent study, Mg/N-modified CQDs coated with FA and HA and conjugated with epirubicin showed higher internalization rates and enhanced cytotoxicity towards 4T1 and MCF-7 cell lines than free drugs. Moreover, experiment on a mouse model revealed that tumor-bearing animals treated with the experimental system suffered the lowest damage to the liver, spleen, and heart, and also presented the highest tumor reduction rate [256].

Owing to the clinical commonness of doxorubicin (DOX) use due to its relatively high anticancer efficacy, and the well-known limitations of this drug, which include cardio-, hepatotoxicity and an increasing rate of resistance acquisition by cancer cells, a considerable number of delivery systems are being designed to increase its efficacy with a subsequent reduction in systemic toxicity. A particularly remarkable case of green-emitting CQD synthesis and testing on BC and healthy cell lines was reported by Li et al. The prepared CQDs used in this study were basically nontoxic, even in the concentration of 100 µg/mL and observation period of 96 h, according to the authors. CQDs were then conjugated with DOX through electrostatic and hydrogen bonding to create a pH-responsive delivery system, which showed selective toxicity towards MCF-7 breast cancer cells with no decline in the viability of healthy cells [257]. The CQD-DOX complex developed by Kong et al. was characterized by higher cellular uptake and better anticancer efficacy on MCF-7 cells than free DOX, as reported in [258]. A similar result was reported for Tf-CQDs loaded with DOX, the uptake rate and cytotoxic efficacy of which significantly exceeded the internalization and toxicity of free DOX, as shown on MCF-7 cells. Moreover, the authors provided the results of molecular docking, evaluating binding energy and site-specific interactions between DOX and Tf [243]. The application of amphiphilic nanomicelle functionalized with FA-coated CQDs and loaded with DOX on MDA-MB-231 cells caused higher cytotoxicity, with IC_50_ value ten times lower in comparison to free DOX under the same conditions, suggesting this system is more efficient, at least, in vitro [259].

The DOX complex with small (~5 nm) GQDs induced a series of events in MCF-7 cells, such as loss of membrane integrity, chromatin condensation with nuclear shrinkage, and fragmentation, leading to apoptosis [260]. GQDs are also shown to improve the DNA cleavage activity of DOX in MCF-7 cells, which is time- and concentration-dependent; it has been highlighted that the key feature enabling the enhancement of DOX-induced DNA double strand breaks by GQDs is the ratio between them and DOX [261]. Another reported approach is coating GQDs with DOX imprinted by a cationic polymer. Such conjugates accumulated in the 4T1 tumor cells of mice, releasing DOX at a relatively slow rate during 72 h of observation. Interestingly, in concentrations ranging between 0.5 and 500 µg/mL, the cytotoxic efficacy of free DOX exceeded the effect of the conjugate; this relation reversed when the concentrations were lowered to 0.05 µg/mL. Although synthesized conjugate did not exhibit remarkable hemolytic properties (about 1% degraded erythrocytes at 0.05 µg/mL), its cytotoxicity towards 4T1 cells did not reach a satisfactory level [262].

Apart from DOX, other chemotherapeutics utilized in BC therapeutic regimens and burdened with high systemic toxicity, such as cisplatin, gemcitabine, or tamoxifen, can be conjugated with QDs in order to create delivery systems. Accordingly, CQDs have been used to construct trackable cisplatin pH/redox dual-responsive carriers with an RGD (Arg-Gly-Asp) peptide as a targeting ligand. Hydrolysis of the benzoic-imine bond, which held the outer PEG layer, in the slightly acidic pH of the TME enabled exposure of the signal peptide, leading to effective uptake by cancer cells through its interaction with integrins. Subsequently, the loaded cisplatin was activated in the cytosol of cancer cells; the model showed anticancer efficacy towards integrin-overexpressing MDA-MB-231 cells [263]. In another study, an EGFR-targeting antibody was attached to the cisplatin-loaded GQDs of pH-dependent release to improve their uptake and toxicity by MDA-MB-231 cells. The role of anti-EGFR was confirmed on cells with saturated EGFR, which exhibited a lower uptake, and therefore the apoptosis-inducing effect of the studied complex was limited [194]. Improvement in cisplatin pharmaceutical performance through its attachment to GQDs was acknowledged by Sui et al. on the basis of increased cancer cell permeability (including the MCF-7 line), enabling more drug to be internalized, induced cell cycle arrest, and enhanced DNA fragmentation upon exposure to such a combined treatment [264].

Recently, successful delivery of tamoxifen using GQD-based nanocarriers with FA as a targeting agent was described in two studies, both reporting a higher cytotoxic activity of the conjugates compared to the drug alone, against breast cancer cells [265,266]. A different targeting molecule, quinic acid, was used to guide gemcitabine-loaded, N-doped CQD complexes towards MCF-7 cells, with some success, as a high accumulation of the conjugates was observed in the cells’ cytoplasm. Quinic acid, apart from its high affinity for angiogenetic factor selectins, presents anticancer activity itself since it is able to induce apoptosis in breast cancer cells [267]. CQDs were also used as gemcitabine carriers [268].

### 6.3. Non-Coding RNA Delivery

Given their abilities to affect gene expression, both si- and miRNAs can be utilized in molecular therapeutic approaches to post-transcriptionally alter the expression of basically any gene of interest. Therefore, it is a promising approach to act on non-druggable targets, such as those that are not accessible or do not show enzymatic functions to be inhibited. Therapeutic strategies involving siRNA entail introducing siRNA into target cells to cause RNA interference and inhibit the expression of a specific mRNA. Conversely, miRNA-based therapies involve two methods. The miRNA inhibition method is similar to antisense therapy, where synthetic single-stranded RNAs serve as miRNA antagonists to block the function of endogenous miRNAs. In the miRNA replacement approach, synthetic miRNAs, referred to as miRNA mimics, replicate the function of endogenous miRNAs, leading to mRNA degradation or inhibition, ultimately causing gene silencing [181].

Since ncRNAs, as indicated before, play tremendous roles in vital cellular processes and can act as both carcinogenesis drivers and cancer suppressors, si- and miRNAs can be applied in cancer therapy through disrupting the expression of oncogenes. Hence, their anticancer abilities are being extensively studied in order to gain the suppression of cancer cell proliferation and stimulation of apoptosis with reduced toxicity as new therapeutic modalities [176,265,269]. Site-specific, efficient intracellular siRNA delivery is challenging for several reasons. Naked RNAs are extremely unstable due to rapid degradation by RNases, resulting in their short lifespan in systems. The negative charge and hydrophilic nature of bare ncRNAs hampers their crossing through plasma membranes; moreover, external ncRNAs can induce immune response. Therefore, the development of carrier systems for ncRNA encapsulation and efficient delivery to cancer cells, where they should accumulate, is required to enable the selective knockdown of genes associated with apoptosis evasion, proliferation, and chemotherapy resistance [270]. Previous studies have proven the ability of siRNAs in reducing breast cancer progression and its metastatic capacity, and I reversing chemotherapy resistance [271].

One such system can be based on QDs, since their use—similar to them being applied in targeted drug delivery—provides an opportunity to simultaneously deliver ncRNAs specifically to cancer cells and track their behavior and route. Although the development of ncRNA-QD conjugates against cancer has attracted high scientific interest, limited preclinical in vitro studies are focused on targeting breast cancer. One of the first reports on this topic concerns the successful delivery of HER2 siRNA by chitosan nanoparticles with encapsulated green fluorescent CdSe/ZnS QDs, functionalized with anti-HER2 antibodies, as shown on SK-BR-3 HER2-overexpressing breast cancer cells and the MCF-7 line [272]. HER2+ breast cancer cells (BT-474 line) were also subjected to transfection with CQD-based complexes with HER3-siRNA and trastuzumab. As HER3 is lacking an intrinsic tyrosine kinase activity, it cannot be targeted with inhibitors of this enzyme; it is, however, an interesting target due to its reported role in mediating anti-HER2 therapy resistance. The conjugate was successful in downregulating HER3 expression, significantly inhibited cell proliferation, and induced apoptosis, while trastuzumab alone caused lower growth suppression [273]. The dual miRNA-triggered drug release system, developed by Yue et al., is based on the ability of miRNA-21 and miRNA-10b, overexpressed in MDA-MB-231 cells, to catalyze the nanocarrier disassembly in an entropy gain process, leading to the release of DOX and two pairs of DNA/RNA duplex, which further hybridize to generate siRNA (siBcl-2) in situ, thereby combining gene therapy and chemotherapy [274].

Using streptavidin-biotin binding, CdSe/ZnS QDs were also used to prepare a carrier for an antisense nucleotide targeting mRNA encoding folate receptor in MCF-7 cells. After the incubation of cells with this agent, upon internalization through receptor-mediated endocytosis, a significant decrease of the folate receptor could be observed [275]. Kim et al. developed an aptamer-coupled cationic nanocarriers containing QDs and siRNAs complementary to Bcl-2 and PKC mRNAs, to achieve a therapeutic effect in EGFR-overexpressing TNBC cells represented by the MDA-MB-231 and MDA-MB-453 cell lines. In this case, QDs did not serve as a delivery vehicle but were incorporated into the lipid bilayer in order to generate a strong fluorescent signal for tracking purposes [276]. Poly(ethyleneimine)-functionalized CQDs have been used to deliver siRNA, targeting VEGF-mRNA to MDA-MB-231 cells and live mice tumors. CQD-based systems have been observed to efficiently protect siRNA from RNase-mediated degradation and allowed their release in the target cell cytoplasm. The used complexes did not negatively affect cell viability, which equaled approximately 90% throughout the experiment, and did not induce immune responses. A significant reduction in VEGF expression was observed, in both cellular and in vivo mouse models [277].

The findings discussed in this chapter are compiled in Table 2.

## 7. Conclusions

QDs have a transformative potential in breast cancer theranostics and can allow one to resolve certain related issues, since their unique physicochemical properties could be used for the earlier detection of BC and the implementation of targeted, personalized therapy. The utilization of QD-based nanoprobes can lead to more accurate diagnosis through the detection of key biomarkers like HER2, CA 15-3, or Ki67, offering a promising alternative to conventional methods. Also, QD–aptamer conjugates for ncRNA detection show great potential, but are still in the early stages of development. Furthermore, their application in targeted drug delivery systems can provide a more effective and personalized treatment approach by minimizing systemic side effects and overcoming drug resistance. This is an area where significant preclinical research has been conducted, making this method the closest to commercialization, as well as widespread future use. However, it still requires significant technological development and research into its long-term safety and efficacy.

Although QDs are under extensive research and considerable efforts are being made to enable their future use in biomedicine, particularly concerning the improvement of cancer diagnosis and treatment, significant limitations and challenges still remain unaddressed. The major issue is related to the in vivo behavior of QD-based delivery vehicles or probes. Apart from ROS generation and possible toxicity, these can interact with cellular components, as well as blood components, interfering with their physiological functions. Still, the impact of QDs on organisms as a whole in real-world exposure scenarios is poorly understood. Maintenance of their stability in biological systems is an additional concern. Ensuring uniform distribution within tumors is also a challenge, as heterogeneous environments such as those of BCs can impede an even distribution of QDs. Obviously, understanding and controlling the biodistribution and clearance mechanisms is essential for minimizing potential long-term risks. Moreover, regulatory and manufacturing issues must be considered. Ensuring consistent and high-quality production of QDs would be essential for their clinical use; the cost-effectiveness and scalability of manufacturing processes are also of importance. However, regulatory approval for medical applications requires comprehensive safety and efficacy data, which can be difficult and costly to obtain for new materials like QDs.

There are many open questions that arise when considering future perspectives and further advances in quantum dot technology, including how to develop standardized protocols for the manufacturing, characterization, and testing of QDs to ensure consistent quality and performance; how to overcome the regulatory barriers for the clinical translation of QD-based technologies; and what choice of strategies/therapeutic agents to combine with QDs will ensure the best anticancer effect, among others. Addressing these open questions requires interdisciplinary collaboration among chemists, biologists, engineers, and clinicians to develop safe, effective, and practical QD-based technologies for cancer diagnosis and treatment.

## Figures and Tables

**Table 1:** Summary of in vitro and animal studies concerning the development of breast cancer modalities based on QDs.

Diagnostic Modality	Type of QDs	Modification/Conjugated Molecule	Size	Targeted Moiety/Ligand/Other	Targeted Cell Line/Organ/Other	Ref.
Antibody-conjugated QD probes	CdSe/ZnS QDs	biotinylated detection antibody	n/a	HER2-ECD	human serum	[164]
	CdS QDs, cysteamine-capped	anti-CA 15-3 antibody	~3 nm	CA 15-3	human serum	[165]
	CuInS_2_/ZnS QDs	anti-Ki-67 monoclonal antibody	n/a	Ki67	MDA-MB-231 and HMMECs	[169]
	InP/ZnS QDs	anti-EGFR antibody	3–5 nm	EGFR	mouse breast cancer xenografts	[189]
	GQDs	single chain variable fragment of anti-EGFR antibody	<5 nm	EGFR	MDA-MB-231	[190]
QD–aptamer/QD–ssDNA conjugates and other miRNA probes	CdTe QDs	ATP-aptamer, mucin-1 aptamer-conjugated gold NPs	2.8 nm	mucin 1 (MUC1)	MCF-7	[172]
	CQDs	complementary single-strand DNA (ssDNA) molecules	3–8 nm	miR-21	MCF-7	[181]
	CdSe/ZnS QDs		9 nm	miR-148, miR-21	MCF-7, MDA-MB-231	[183]
	Antimonide QDs	n/a	n/a	miR-21, miR-155	human serum	[184]
	AuNPs/GQDs/GO	miRNA capture probes	n/a	miR-21, miR-155, miR-210	human serum	[185]
Other	N-doped GQDs	phytohemagglutinin-L (PHA-L)	3–12 nm	PHA-L	MCF-7	[173]
	N-doped GQDs	folic acid capping	GQDs: 10 nm, conjugate: 15 nm	folate receptor (FR)	MKN-45, HT-29, MCF-7	[194]

**Table 2:** Summary of in vitro and animal studies concerning the development of breast cancer therapeutic modalities based on QDs.

Therapeutic Modality	Type of QDs Used	Conjugated Molecule	Size	Cell Line/Model	Role of QDs	Ref.
Photothermal therapy	tea polyphenol-derived CQDs	-	1.3–3.7 nm	mouse xenografts of 4T1 cells	type I PS	[232]
	polythiophene-derived GQDs	-	2–6 nm	mouse breast cancer xenografts	type II PS	[233]
	N-doped GQDs/TiO_2_ NCs	-	9.16 ± 2.4 nm	MDA-MB-231 and HS27	type I/II PS	[234]
	ZnO QDs	-	n/a	MDA-MB-231	type I PS	[237]
Photodynamic therapy	CQD clathrates	MTX	20–30 nm	HMLER (breast cancer stem cells)	PS and drug carrier	[235]
	InP/ZnS QDs	chlorin e6	2–3 nm	MDA-MB-231	carrier of a PS, enhancer of energy transfer	[236]
Targeted drug delivery	Mg/N-modified CQDs, FA/HA-coated	epirubicin	6–7 nm	4T1, MCF-7	delivery vehicles of chemotherapeutics to the tumor site, increasing their efficacy *	[252]
	CQDs	DOX	2–6 nm	MCF-7		[253]
	CQDs	DOX	CQDs: 0.5 nm, conjugates: 20 nm	MCF-7		[254]
	Tf-CQDs	DOX	1.5 nm	MCF-7		[243]
	FA-coated CQDs	DOX	1.5–8 nm	MDA-MB-231		[255]
	GQDs	DOX	5 nm	MCF-7		[256]
	GQDs-cationic polymer	DOX	conjugates: <55 nm	mouse xenografts of 4T1 cells		[258]
	CQDs	cisplatin	5–8 nm	MDA-MB-231		[259]
	GQDs	cisplatin, anti-EGFR antibody	<5 nm	MDA-MB-231		[194]
	GQDs	cisplatin	40 nm	MCF-7		[260]
	FA-coated, PEGylated GQDs	tamoxifen	50–210 nm	MCF-7		[262]
	quinic acid-coated N-doped GQDs	gemcitabine	n/a	MCF-7		[263]
Non-coding RNA/aptamer delivery	CdSe/ZnS QDs	HER2 siRNA, anti-HER2 antibodies	conjugates: 60–80 nm	SK-BR-3, MCF-7	siRNA/drug delivery vehicles	[268]
	CQDs	HER3 siRNA, trastuzumab	4 nm	BT-474		[269]
	CdTe QDs	DOX, Bcl-2 siRNA	QDs: 26 nm, conjugates: 90 nm	MDA-MB-231		[270]
	CdSe/ZnS QDs	folate receptor siRNA	~15 nm	MCF-7		[271]
	n/a	Bcl-2 and PKC siRNAs	n/a	MDA-MB-231, MDA-MB-453	fluorescence generator for tracking purposes	[272]
	PEI-CQDs	VEGF siRNA	3–7 nm	MDA-MB-231, mouse xenografts	siRNA delivery vehicles, protection from RNAses	[273]

* This is a general statement. Not in all studies, the superior efficacy of QD-containing construct over the free drug has been confirmed (refer to the main text for details).

## Data Availability

Not applicable.
